# Modeling photosynthetic resource allocation connects physiology with evolutionary environments

**DOI:** 10.1038/s41598-021-94903-0

**Published:** 2021-08-05

**Authors:** Esther M. Sundermann, Martin J. Lercher, David Heckmann

**Affiliations:** Institute for Computer Science and Department of Biology, Heinrich Heine University Düsseldorf, 40225 Düsseldorf, Germany

**Keywords:** Computational models, Evolution, C4 photosynthesis, Plant evolution

## Abstract

The regulation of resource allocation in biological systems observed today is the cumulative result of natural selection in ancestral and recent environments. To what extent are observed resource allocation patterns in different photosynthetic types optimally adapted to current conditions, and to what extent do they reflect ancestral environments? Here, we explore these questions for C_3_, C_4_, and C_3_–C_4_ intermediate plants of the model genus *Flaveria*. We developed a detailed mathematical model of carbon fixation, which accounts for various environmental parameters and for energy and nitrogen partitioning across photosynthetic components. This allows us to assess environment-dependent plant physiology and performance as a function of resource allocation patterns. Models of C_4_ plants optimized for conditions experienced by evolutionary ancestors perform better than models accounting for experimental growth conditions, indicating low phenotypic plasticity. Supporting this interpretation, the model predicts that C_4_ species need to re-allocate more nitrogen between photosynthetic components than C_3_ species to adapt to new environments. We thus hypothesize that observed resource distribution patterns in C_4_ plants still reflect optimality in ancestral environments, allowing the quantitative inference of these environments from today’s plants. Our work allows us to quantify environmental effects on photosynthetic resource allocation and performance in the light of evolutionary history.

## Introduction

Metabolic efficiency is an important determinant of organismal fitness^1,2^. Major constraints on metabolic fluxes can arise from scarcity of chemical compounds, e.g., nitrogen necessary to produce enzymes^3^, or from the limited solvent capacity of cellular compartments^4,5^. To ensure optimal metabolic efficiency, gene regulation has to balance available resources appropriately. Modern methods of modeling metabolism rely strongly on the assumption of metabolic optimality under physicochemical constraints^6–8^. Accordingly, resource allocation and its constraints are under intense investigation, although these studies are mostly restricted to unicellular organisms. However, the metabolic efficiency of a given metabolic system is not static, but depends on the environment. Thus, uncertainties about the environmental properties that an organism has adapted to remain a major obstacle in the application of these methods. Autotrophic systems, such as plant leaves, are ideal to study the interaction of the environment and resource allocation, as the diversity of nutrient sources is much lower than for heterotrophs, which results in a reduced complexity of the space of possible environments. Furthermore, the effect of environmental factors on plant performance, e.g., the rate of CO_2_ assimilation, have been studied intensively^9^. In particular, C_3_ and C_4_ photosynthesis represent complementary gene expression and resource allocation patterns that result in high fitness in specific ecological niches.

In all plants, the fixation of carbon from CO_2_ is catalyzed by the enzyme ribulose-1,5-bisphosphate carboxylase/oxygenase (Rubisco) as part of the Calvin-Benson cycle. Rubisco also shows an affinity for O_2_, resulting in a toxic by-product, which needs to be recycled by the photorespiratory pathway and causes a significant loss of carbon and energy^10^. Rubisco is an important resource sink in the leaf proteome of plants: it utilizes up to 30% of leaf nitrogen and up to 65% of total soluble protein^11,12^. While C_3_ plants operate the Calvin-Benson cycle in their mesophyll cells to fix carbon, C_4_ plants express it in the bundle sheath cells and use phospho*enol*pyruvate (PEP) carboxylase (PEPC) in their mesophyll cells for the initial fixation of carbon. The resulting C_4_ acids are eventually decarboxylated in the bundle sheath cells, creating a local high-CO_2_ environment around Rubisco that suppresses photorespiration. While the exact biochemical implementation of the C_4_ cycle varies between species, the C_4_ cycle is completed by the regeneration of PEP by pyruvate, phosphate dikinase (PPDK).

Compared to C_3_ photosynthesis, C_4_ metabolism requires additional nitrogen to produce the C_4_ enzymes; this additional investment is counteracted by reduced Rubisco requirements due to the concentration of CO_2_ around Rubisco^13^. The energy requirements of C_4_ metabolism also differ from those of the C_3_ pathway^14^, as further ATP is needed for the regeneration of PEP, while ATP and NADPH requirements of the photorespiratory pathway are reduced. The metabolic efficiencies of the C_3_ and C_4_ system depend strongly on the environment. To achieve optimal metabolic efficiency, plants have to coordinate gene expression of the Calvin-Benson cycle, photorespiration, light reactions, and, in the case of C_4_ plants, the C_4_ cycle; this coordination occurs in a complex response to the availability of light energy and nitrogen and of factors that influence the rate of photorespiration. The diversity of photosynthetic resource allocation patterns is emphasized by the existence of C_3_–C_4_ intermediate photosynthesis in some plants, where features of the archetypical C_4_ syndrome are only partially expressed. The genus *Flaveria* contains closely related species that employ the C_3_, C_3_–C_4_ intermediate, and C_4_ versions of photosynthetic metabolism, making it an ideal system to study the interaction between resource allocation and environment in photosynthesis.

The selection pressures caused by environmental factors over evolutionary time scales are expected to lead to corresponding adaptations of gene regulation. In contrast, environmental variation on the time scale of individual generations may select for regulatory programs that adjust plant metabolism to the environment they currently face, a process called phenotypic plasticity. Reviewing the occurrence of phenotypic plasticity in C_3_ and C_4_ plants, Sage and McKown^15^ argued that C_4_ plants show limited regulation of Rubisco content in response to environmental factors like sunflecks and low temperatures. Although the extent of phenotypic plasticity in plants is intensively studied e.g.^15–18^, the plasticity in terms of resource allocation is not fully understood. In particular, it is not clear whether the phenotypic plasticity of different plant lineages is sufficient to acclimate optimally to the current environment; instead, many plants might still allocate at least parts of their resources in patterns that were optimal in the environments that dominated their recent evolutionary history.

The areas where C_4_ dicotyledonous plants are assumed to have evolved are regions of low latitude showing combinations of heat, drought, and salinity^13^. For *Flaveria*, analyses that combine phylogenetic context and environmental information point toward an evolutionary origin in open habitats with high temperatures^13,19,20^. The last common C_3_ ancestor of the current *Flaveria* species lived 2–3 million years ago^21^, when CO_2_ levels were significantly lower than the current, postindustrial level^22,23^. In summary, *Flaveria* species likely faced high light intensities, high temperatures, and low atmospheric CO_2_ levels during their recent evolutionary history.

The standard method to model the CO_2_ assimilation rate of C_3_, C_4_, and C_3_–C_4_ intermediate plants is based on the mechanistic biochemical models of Berry and Farquhar^24^, Farquhar et al.^25^, and von Caemmerer^9,26^. These models predict the light- and enzyme-limited CO_2_ assimilation rate with great success, and take into consideration enzymatic activities and various environmental parameters, including mesophyll CO_2_ level and light intensities. In many ecosystems, the most limiting resource for plant growth is nitrogen^27,28^, and a high proportion of nitrogen is used in photosynthesis^29^. The increased nitrogen-use efficiency of C_4_ species compared to C_3_ relatives indicates that nitrogen availability may have played a major role in C_4_ evolution^30^. Models of optimal nitrogen allocation were successfully used to understand the response to environmental factors like elevated CO_2_^31,32^, light^33,34^, and temperature^35^, but these approaches were limited to C_3_ plants. In order to understand how optimal resource allocation patterns shifted during C_4_ evolution, a new modeling framework is required.

Here, we aim for a detailed understanding of the interplay between resource allocation and current and past evolutionary environments in relation to CO_2_ assimilation occurring in C_3_, C_4_, and C_3_–C_4_ intermediate species. To achieve this goal, we developed a mathematical model for these photosynthetic types that integrates knowledge of resource costs and relevant environmental factors. Using this model, we seek to understand (1) to what extent resource allocation is phenotypically plastic and to what extent it appears adapted to an environment the plants were facing during their evolutionary history; and (2) if resource allocation patterns can be used to identify unique environments to which allocation is optimally adapted.

## Methods

### Model overview

**Figure 1:**
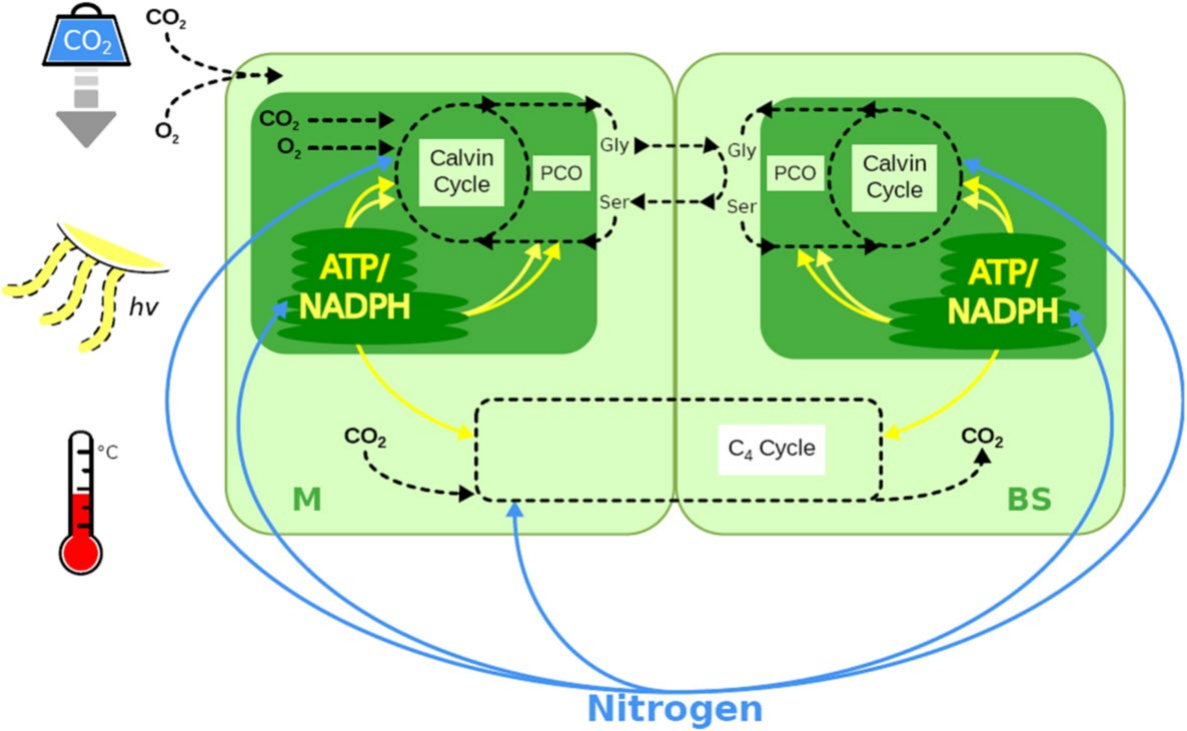
An overview of the nitrogen-dependent light- and enzyme-limited model. CO_2_ entering the mesophyll cell (M) can be fixed by Rubisco (C_3_ and intermediates) or PEPC (C_4_ and intermediates); The C_4_ cycle then shuttles CO_2_ fixed by PEPC to the bundle sheath cell (BS) and releases it, allowing it to be re-fixed by Rubisco. The fixation of O_2_ by Rubisco leads to photorespiration (PCO). Blue arrows indicate the nitrogen allocation and yellow arrows represent the energy allocation considered in the model.

Here, we present a nitrogen-dependent light- and enzyme-limited model for the steady-state CO_2_ assimilation rate, which—depending on its parameterization—can describe C_3_, C_3_–C_4_ intermediate, and C_4_ photosynthetic types. Figure 1 shows a schematic overview, highlighting the relationships between the major pools of photosynthetic nitrogen (Rubisco, C_4_ cycle, and thylakoids). The definitions of the corresponding model parameters are listed in Table 1. Not all parameters are represented explicitly in Fig. 1, e.g., the schematic figure does not distinguish linear and cyclic electron transport, or the two enzymes PEPC and PPDK that represent the C_4_ cycle. In this study, we parameterize the model to describe species from the genus *Flaveria;* parameter values are listed in Supplementary Table S1. Before describing the model components in detail, we provide an overview of the model in the following paragraphs.

**Table 1:** A list of all parameters used in the mathematical model.

Abbrev	Explanation	Value	Units
*A*	Achieved CO_2_ assimilation rate $(A=\mathrm{ min}\left({A}_{j}, {A}_{c}\right))$		µmol m^−2^ s^−1^
*A*_*c*_	CO_2_ assimilation rate when the electron transport rate is not limiting		µmol m^−2^ s^−1^
*A*_*j*_	Light-limited CO_2_ assimilation rate ${(A}_{j}=\mathrm{min}\left({A}_{j}^{ATP}, {A}_{j}^{NADPH}\right))$		µmol m^−2^ s^−1^
${A}_{j}^{ATP}$	ATP-limited CO_2_ assimilation rate		µmol m^−2^ s^−1^
${A}_{j}^{NADPH}$	NADPH-limited CO_2_ assimilation rate		µmol m^−2^ s^−1^
${\mathrm{c}}_{\mathrm{E}}$	Number of Rubisco catalytic sites per nitrogen	1.27 × 10^–3^ ^36^	µmol × (µmol nitrogen)^−1^
*c*_*N*_	Nitrogen costs of cytochrome f	8.85^49^	mol nitrogen × (mmol cyt)^−1^
*Chl*	Chlorophyll content		µmol m^−2^
*cyt*	The amount of cytochrome f per chlorophyll		mmol × (mol Chl)^−1^
*cyt*_*Jmax*_	The relation of cytochrome f to *J*_*max*_	172^50^	mmol electron × (mmol cyt s)^−1^
*e*_*ATP*_	Assumed ratio of electron per ATP in the linear electron transport	4/3^66^	electron × ATP^−1^
*E*_*tot*_	The amount of Rubisco catalytic sites		µmol m^−2^
*f*	A correction factor accounting for the spectral quality of the light	0.15^9^	unitless
*g*_*s*_	The bundle sheath cell conductance		µmol m^−2^ s^−1^
*I*	The absorbed light		µmol m^−2^ s^−1^
*J*_*max*_	The maximal electron transport rate		µmol m^−2^ s^−1^
*Jmax*_*CET*_	The maximal electron transport rate of the cyclic electron transport		µmol m^−2^ s^−1^
*Jmax*_*CL*_	A factor that describes the scaling of maximal electron transport rate with cytochrome f for the CET	3 (assumption)	factor
*Jmax*_*LET*_	The maximal electron transport rate of the linear electron transport		µmol m^−2^ s^−1^
*J*_*t*_	Electron transport rate		µmol m^−2^ s^−1^
*J*_*mc*_	Electron transport rate that is available for the Calvin-Benson cycle and the photorespiratory path in the mesophyll cell		µmol m^−2^ s^−1^
*J*_*mp*_,	Electron transport rate that is available for the C_4_ cycle		µmol m^−2^ s^−1^
*J*_*s*_	Electron transport rate that is available for the Calvin-Benson cycle and the photorespiratory path in the bundle sheath cell		µmol m^−2^ s^−1^
*I*_*CET*_	Irradiance absorbed by the pigments of the cyclic electron transport		µmol m^−2^ s^−1^
*I*_*LET*_	Irradiance absorbed by the pigments of the linear electron transport		µmol m^−2^ s^−1^
*k*_*ccat*_	Turn-over rate of Rubisco		s^−1^
*k*_*cat﻿,PEPC*_	Turn-over rate of PEPC	66^46^	s^−1^
*k*_*c﻿at,PPDK*_	Turn-over rate of PPDK	6.02^46^	s^−1^
*K*_*p*_	Michaelis constant of PEPC for bicarbonate		µbar
*LHC*	Light harvesting complexes		mmol × (mol Chl)^−1^
*MW*_*PEPC*_	The nitrogen requirement of a catalytic site of PEPC	96,000^46^	Da
*MW*_*PPDK*_	The nitrogen requirement of a catalytic site of PPDK	95,000^46^	Da
*n*_*C4*_	The fraction of photosynthetic nitrogen pool invested into the main enzymes of the C_4_ cycle: PEPC and PPDK		fraction
${n}_{C4}^{evo}$	The optimal fraction of photosynthetic nitrogen pool invested into the main enzymes of the C_4_ cycle under the evolutionary scenario		fraction
${n}_{C4}^{growth}$	The optimal fraction of photosynthetic nitrogen pool invested into the main enzymes of the C_4_ cycle under the growth scenario		fraction
*n*_*Chl*_	Empirical factor that relates the amount of nitrogen invested into thylakoids to the amount of chlorophyll in C_3_ plants	0.0158887^39^	factor
*n*_*Etot*_	The fraction of photosynthetic nitrogen pool invested into the Calvin-Benson cycle		fraction
${n}_{Etot}^{evo}$	The optimal fraction of photosynthetic nitrogen pool invested into the Calvin-Benson cycle under the evolutionary scenario		fraction
${n}_{Etot}^{growth}$	The optimal fraction of photosynthetic nitrogen pool invested into the Calvin-Benson cycle under the growth scenario		fraction
*n*_*fit*_	The proportion of nitrogen invested into the thylakoids as a function of the leaf nitrogen level (a fit to empirical data)		fraction
*n*_*Jmax*_	The fraction of photosynthetic nitrogen pool invested into the thylakoids, which include the electron transport chains		fraction
${n}_{Jmax}^{evo}$	The optimal fraction of photosynthetic nitrogen pool invested into the thylakoids under the evolutionary scenario		fraction
${n}_{Jmax}^{growth}$	The optimal fraction of photosynthetic nitrogen pool invested into the thylakoids under the growth scenario		fraction
*n*_*Rubisco*_	Empirical nitrogen investment of C_3_ *Flaveria* species into Rubisco		fraction
*N*_*ps*_	Photosynthetic nitrogen pool		µmol m^−2^
*N*_*t*_	Total leaf nitrogen		µmol m^−2^
*N*_*thy*_	Nitrogen invested into the thylakoids		µmol × (µmol Chl)^−1^
*p*	proportion of linear electron transport		fraction
*PSI*	Photosystem I	2^49^	mmol × (mol Chl)^−1^
*PSI*_*CET*_	Photosystem I that is associated with the cyclic electron transport		mmol × (mol Chl)^−1^
*PSI*_*LET*_	Photosystem I that is associated with the linear electron transport		mmol × (mol Chl)^−1^
*PSII*	Photosystem II	2.5^49^	mmol × (mol Chl)^−1^
*pI*_*Chl*_	Mol chlorophyll per mol complex of PSI	184^49^	mol Chl × (mol complex)^−1^
*pII*_*Chl*_	Mol chlorophyll per mol complex of PSII	60^49^	mol Chl × (mol complex)^−1^
*pI*_*N*_	Nitrogen costs of PSI	32.8^49^	mol nitrogen × (mol Chl)^−1^
*pII*_*N*_	Nitrogen costs of PSII	83.3^49^	mol nitrogen × (mol Chl)^−1^
*l*_*Chl*_	Mol chlorophyll per mol complex of LHC	13^49^	mol Chl × (mol complex)^−1^
*l*_*N*_	Nitrogen costs of the LHC	26^49^	mol nitrogen × (mol Chl)^−1^
*V*_*pmax*_	Maximal C_4_ cycle activity		µmol m^−2^ s^−1^
*α*	Leaf absorptance	0.84^9^	fraction
β	Rubisco distribution between mesophyll and bundle sheath cells		fraction
*δ*_*n*_	Required nitrogen re-allocation		fraction
*Θ*	The convexity of the transition between the initial slope and the plateau of the hyperbola	0.7^9^	unitless
*ξ*	The fraction of glycine decarboxylated in the bundle sheath cell that is derived from oxygenation by Rubisco in the mesophyll cell		fraction

For each parameter, we list abbreviation (abbrev.), explanation, default value (if this exists, with reference) and units.

We extended the light- and enzyme-limited C_3_–C_4_ models originally developed by von Caemmerer^9^ and modified by Heckmann, et al.^2^. We added a fixed budget of nitrogen constraining the total abundance of photosynthetic proteins using previous knowledge about the major nitrogen requirements of photosynthetic components, e.g., Rubisco^36^. Furthermore, we extended the existing models by explicitly modeling the ATP and NADPH production of the linear and cyclic electron transport (LET and CET, respectively). Thus, an environment-dependent photosynthetic nitrogen budget is distributed across the enzymes of the Calvin-Benson cycle in the mesophyll and bundle sheath cell, the C_4_ cycle, and the proteins of the LET and CET in the thylakoid membranes. Combining this model with the temperature dependency of the photosynthetic apparatus^37^ results in a detailed model of photosynthesis that incorporates leaf nitrogen level, light intensity, mesophyll CO_2_ and O_2_ levels, and the effects of temperature.

In order to understand physiological data in the context of environmental adaptation, we aim to find optimal resource allocation in a given environment. To this end, we assume that resource allocation has been optimized by natural selection to maximize the CO_2_ assimilation rate (*A*, [µmol m^−2^ s^−1^])^23,38,39^. We developed a robust optimization pipeline that reliably finds optimal resource allocation dependent on environments and photosynthetic types. In previous work, optimality assumptions were successfully used in a variety of photosynthetic systems; examples are the explanation of the coordination of ribulose-1,5-bisphosphate carboxylation and regeneration during C_3_ photosynthesis^40,41^, optimal nitrogen allocation in C_3_ plants in different environments^31–35,42^, the exploration of evolutionary trajectories from C_3_ to C_4_ photosynthesis^2^, the exploration of alternative inter-cellular transport pathways in C_2_ plants^43^, and the prediction of proteome allocation in cyanobacteria^44^.

We use optimality of the modeled CO_2_ fixation rate to determine (1) the optimal relative investment of nitrogen into Rubisco, the C_4_ cycle enzymes, and the proteins of the light-dependent reactions, (2) the optimal partitioning of NADPH between the Calvin-Benson cycle and the photorespiratory pathway, (3) the optimal partitioning of ATP across the Calvin-Benson cycle, photorespiratory pathway, and C_4_ cycle (if relevant), and (4) the optimal proportion of LET and CET.

### Environmental factors and evolutionary parameters

We specify the environment in terms of the following factors: light intensity, leaf nitrogen level, temperature, and CO_2_ and O_2_ mesophyll partial pressures. The photosynthetic type is defined by six parameters: the Rubisco distribution between mesophyll and bundle sheath cells (β); the Rubisco kinetics, (specified through a single parameter, *k*_*ccat*_ [s^−1^], due to the known trade-off relationships between the kinetic parameters^45^); the maximal C_4_ cycle activity (*V*_*pmax*_, [µmol m^−2^ s^−1^]); the fraction of glycine decarboxylated by the glycine decarboxylase complex in the bundle sheath cell that is derived from oxygenation by Rubisco in the mesophyll cell (*ξ*); the Michaelis constant of PEPC for bicarbonate (*K*_*p*_, [µbar]), and the bundle sheath cell conductance for CO_2_ (*g*_*s*_, [µmol m^−2^ s^−1^]) (see Heckmann, et al.^2^ for details). The values for the parameters are taken from the literature (see Supplementary Table S1 for details).

### Nitrogen allocation

To calculate the CO_2_ assimilation rate, we focus on the photosynthetic nitrogen pool (*N*_*ps*_, [µmol m^−2^]). In our model, *N*_*ps*_ can be allocated across the following major pools of leaf photosynthetic nitrogen: the main enzyme of the Calvin-Benson cycle (*n*_*Etot*_*)*, Rubisco; the main enzymes of the C_4_ cycle (*n*_*C4*_), PEPC and PPDK (we decided to focus on PEPC and PPDK as the major nitrogen pools of the C_4_ cycle based on the enzyme molecular weights and turnover numbers^46^); and the thylakoids (*n*_*Jmax*_), which include the photosynthetic electron transport chains. The CO_2_ assimilation rate and other model parameters can be predicted for a freely chosen nitrogen allocation. Note that we are interested in determining the optimal nitrogen allocation (see Section “Optimization procedure” for details). The environment-specific *N*_*ps*_ is calculated as a fraction of total leaf nitrogen (*N*_*t*_, [µmol m^−2^]) based on phenomenological observations (see Supplementary Methods S1 for details).

#### Nitrogen allocated to Rubisco

We only consider the nitrogen requirements of Rubisco in the Calvin-Benson cycle, as it accounts for the major nitrogen costs of this cycle^47^. The amount of catalytic sites of Rubisco (*E*_*tot*_, [µmol m^−2^]) is calculated from the invested nitrogen by Eq. (1), where *n*_*Etot*_ represents the fraction of *N*_*ps*_ invested into Rubisco:

1 $${E}_{tot}={\mathrm{n}}_{Etot} \cdot {N}_{ps} \cdot {\mathrm{c}}_{\mathrm{E}},$$

The number of catalytic sites per nitrogen is 1.27 × 10^–3^ [${\mathrm{c}}_{\mathrm{E}},$ µmol catalytic sites (µmol nitrogen)^−1^] and was derived from Harrison et al. ^36^.

#### Nitrogen allocated to enzymes of the C_4_ cycle

We calculated the nitrogen cost of C_4_ cycle enzymes from data on enzyme kinetics. The nitrogen requirements of the C_4_ cycle consider co-limitation of PEPC and PPDK, whose molecular weights (MW, [Da]) and turn-over rates (*k*_*cat*_, [s^−1^]) are used to calculate the maximal rate of C_4_ cycle activity^46,48^. Equation 2 represents the relationship between the maximal turnover rate, *V*_*pmax*_, and nitrogen investment into the C_4_ enzymes (*n*_*C*4 _*N*_*ps*_),

2 $${V}_{pmax}=\frac{{{n}_{C4} \cdot N}_{ps} }{\begin{array}{c}\left(\frac{{{MW}^{*}}_{PPDK}}{{k}_{cat,PPDK}}\right)+\left(\frac{{{MW}^{*}}_{PEPC}}{{k}_{cat,PEPC}}\right)\end{array}}$$

where MW* ([Da]) represents the nitrogen requirement of a catalytic site, assuming the nitrogen content of the protein is 16%^11^; indices indicate the considered enzyme.

#### Nitrogen and the maximal electron transport rate

Nitrogen invested into the thylakoids (*N*_*thy*_, [µmol (µmol Chl)^−1^]) is related to the maximal electron transport rate (*J*_*max*_, [µmol m^−2^ s^−1^]) via the amount of cytochrome f per chlorophyl (cyt, [mmol (mol Chl)^−1^]) and by considering photosystems I and II (PSI and PSII, [mmol (mol Chl)^−1^]) as well as the light harvesting complexes (LHC, [mmol (mol Chl)^−1^]). In the following, we describe these relationships in quantitative detail [Eqs. (3)–(15)]; indices represent the considered pathway:

3 $$PS{I}_{LET}=2\cdot p$$

4 $$PS{I}_{CET}=2\cdot \left(1-p\right)$$

5 $$PSII=2.5$$

6 $${LHC}_{LET}=\frac{1000 \cdot p-PSII \cdot pI{I}_{Chl}-PS{I}_{LET} \cdot p{I}_{Chl}}{{l}_{Chl}}$$

7 $${LHC}_{CET}=\frac{1000 \cdot \left(1-p\right)-PS{I}_{CET} \cdot p{I}_{Chl}}{{l}_{Chl}}$$

We use previous knowledge about the relationship of thylakoid nitrogen costs and cyt as well as data from Ghannoum et al.^49^ for abundances of PSI and PSII to include phenomenological stoichiometry rules between LHC and the components of the electron transport chain [Eqs. (3)–(7)]; *pII*_*Chl*_, *pI*_*Chl*_, and *l*_*Chl*_ represent mol Chl (mol complex)^−1^ for PSII, PSI, and LHC, respectively). While we parameterize our model for *Flaveria*, the data of Ghannoum et al.^49^ is for C_4_ grasses and for C_3_ dicots; however, as the data was very similar between the diverse species examined, it is likely that values in *Flaveria* are very similar. We assume that the chlorophyll content is shared between PSI, PSII, and LHC [Eqs. (6), (7)]. We extended the previous work by splitting these complexes according to the proportion of LET (*p*) and CET (1—*p*).

For the LET, *J*_*max*_ is related to *N*_*thy*_ as described in Eqs. (8)–(11). ${{N}_{thy}}_{LET}$ represents the available amount of nitrogen for the thylakoids with *n*_*Jmax*_ representing the fraction of photosynthetic nitrogen pool invested into the thylakoids [Eq. (8)], accounting for LHC, PSII, PSI, and cyt [Eqs. (9), (10)]. The amount of cyt can be calculated according to Eq. (10) and related to *J*_*max*_ via the empirical *cyt*_*Jmax*_; *cyt*_*Jmax*_ describes the relation of cyt to *J*_*max*_ and was measured by Niinemets and Tenhunen^50^, who determined 156 mmol e^-^ (mmol cyt s)^−1^ across various C_3_ species. We are not aware of a comparable data set for C_4_ plants. Assuming 95% of LET in C_3_ plants, this leads to a capacity of 172 mmol e^-^ (mmol cyt s)^−1^ for *cyt*_*Jmax*_.

8 $${{N}_{thy}}_{LET}=\frac{{n}_{Jmax}{ \cdot N}_{ps} \cdot p}{Chl}$$

9 $${{N}_{LH}}_{LET}=PSII\cdot {pII}_{N}\cdot {pII}_{Chl}\cdot {10}^{-3}+PS{I}_{LET}\cdot {pI}_{N}\cdot {pI}_{Chl}\cdot {10}^{-3}+LH{C}_{LET}\cdot {l}_{N}\cdot {l}_{Chl}\cdot {10}^{-3}$$

10 $$cy{t}_{LET}=\frac{1}{{\mathrm{c}}_{\mathrm{N}}}\left({N}_{th{y}_{LET}}-{N}_{L{H}_{LET}}\right)$$

11 $$Jma{x}_{LET}=\mathrm{max}\left(0,\frac{cy{t}_{LET} \cdot Chl \cdot cy{t}_{Jmax}}{1000}\right)$$

Chlorophyll content (*Chl*, [µmol m^−2^]) is calculated based on an empirical factor^39^ that relates the amount of nitrogen invested into thylakoids to the amount of chlorophyll in C_3_ plants (see Supplementary Methods S2 for details). We again use work from Ghannoum et al.^49^ to relate *N*_*thy*_ to the amount of cyt [Eqs. (8)–(10)]; *c*_*N*_ represents mol nitrogen per mmol cyt, and *pII*_*N*_, *pI*_*N*_, and *l*_*N*_ represent mol nitrogen per mol Chl for PSII, PSI, and LHC, respectively).

The derivation for the CET is analogous to the case of the LET:

12 $${N}_{th{y}_{CET}}=\frac{{n}_{Jmax} \cdot {N}_{ps} \cdot \left(1-p\right)}{Chl}$$

13 $${{N}_{LH}}_{CET}=PS{I}_{CET}\cdot {pI}_{N}\cdot {pI}_{Chl}\cdot {10}^{-3}+LH{C}_{CET}\cdot {l}_{N}\cdot {l}_{Chl}\cdot {10}^{-3}$$

14 $$cy{t}_{CET}=\frac{1}{{c}_{N}}\left({N}_{th{y}_{CET}}-{N}_{L{H}_{CET}}\right)$$

15 $$Jma{x}_{CET}=\mathrm{max}\left(0,\frac{cy{t}_{CET} \cdot Chl \cdot cy{t}_{Jmax} \cdot Jma{x}_{CL}}{1000}\right),$$

in the last equation, we additionally required the factor *Jmax*_*CL*_, which describes the scaling of *J*_*max*_ with cyt for the CET. This factor is assumed to be 3, as PSII is more expensive in terms of nitrogen compared to PSI^47,49^.

In summary, the free optimization parameters related to nitrogen allocation to the light reactions, *p* and *n*_*Jmax*_, affect *J*_*max*_ in LET and CET via the cytochrome f content.

### Optimization procedure

Theoretically, model predictions can be made using a freely chosen resource allocation. To understand the raised questions about environmental adaptation, we will analyze the fittest plants, i.e., plants with the resource allocation that results in the maximal CO_2_ assimilation rate. To find the maximal CO_2_ assimilation rate under the given environmental, physiological, and biochemical constraints, we optimize the allocation of photosynthetic nitrogen (assumed to depend only on total leaf nitrogen) into Rubisco (*n*_*Etot*_), C_4_ cycle (*n*_*C4*_), LET, and CET (the latter two represented by *p* and *n*_*Jmax*_) through an augmented Lagrangian approach using the auglag-function of the package ‘nloptr’^51^. The optimization is constrained to make sure that the results are biologically realistic with respect to the modeled photosynthetic type, e.g., C_3_ species were not able to invest nitrogen into the C_4_ cycle (see Supplementary Table S2 for details). The model and its optimization were implemented in the R environment^52^ (see Supplementary Methods S3 for details).

### Modeling the effect of light

The relationship of the electron transport rate (*J*_*t*_, [µmol m^−2^ s^−1^]) and the absorbed light of a certain irradiance (*I*, [µmol m^−2^ s^−1^]) is presented in Eqs. (16)–(18). *I* is related to *J*_*t*_ by a widely accepted empirical hyperbolic function [Eq. (16)], ^9,53^ that includes the following parameters: (1) *J*_*max*_, the maximum electron transport rate; (2) *Θ*, the convexity of the transition between the initial slope and the plateau of the hyperbola; (3) *α*, the leaf absorptance; (4) *f*, a correction factor accounting for the spectral quality of the light; and (5) *p,* the fraction of absorbed quanta that reaches PSI and PSII of LET (with (1 − *p*) reaching the CET). *I*_*abso*_ is set to either *I*_*LET*_ or *I*_*CET*_ depending on the considered path of electron transport. The fraction of irradiance that is absorbed by the LET is shared equally between PSI and PSII [resulting in the factor 0.5 in Eq. (17)], while the fraction of irradiance that is absorbed by the CET is assumed to reach PSI in full.

16 $${J}_{t}=\frac{{I}_{abso}+{J}_{max}-\sqrt{{\left({I}_{abso}+{J}_{max}\right)}^{2}-4 \theta {I}_{abso}{J}_{max}}}{2\theta }$$

17 $${I}_{LET}= I\cdot \alpha \cdot \left(1-f\right) \cdot p\cdot 0.5$$

18 $${I}_{CET}= I\cdot \alpha \cdot \left(1-f\right)\cdot \left(1-p\right)$$

In our model it is assumed that the electron transport chain is the only source of ATP and NADPH and that both are used exclusively for CO_2_ fixation^9^. As NADPH production results from LET, the amount of electrons is calculated using Eqs. (16) and (18). The amount of electrons utilized for ATP production depends on both LET and CET. There are multiple pathways of CET^55^; the model considers those pathways with an active Q-cycle and a ratio of two protons per electron. Note that Rubisco is assumed to be fully activated, independent of the irradiance^9^.

The available energy needs to be partitioned between five pools: (1) the Calvin-Benson cycle in the mesophyll; (2) the Calvin-Benson cycle in the bundle sheath; (3) the photorespiratory pathway in the mesophyll; (4) the photorespiratory pathway in the bundle sheath cell; and (5) the C_4_ pathway. This means that the available energy is calculated in total and then partitioned^54^ into *J*_*mp*_, *J*_*mc*_*,* and *J*_*s*_, the fractions of *J*_*max*_ invested into the C_4_ cycle, the Calvin-Benson cycle and the photorespiratory pathway in the mesophyll, and the Calvin-Benson cycle and the photorespiratory pathway in the bundle sheath cell, respectively. During optimization, the activity of each process is constrained by its allocated energy pool, i.e., the energy allocation equals the relative energy allocation of the processes (see Supplementary Methods S3 for details). In summary, the optimal energy allocation is a function of the nitrogen pools.

### CO_2_ assimilation rate

A limitation in the production of both ATP and NADPH arises under light-limited conditions^9^. The ATP-limited (${A}_{j}^{ATP}$) and the NADPH-limited (${A}_{j}^{NADPH}$) CO_2_ assimilation rate are calculated according to the light-limiting model of von Caemmerer^9^ (see Supplementary Methods S5 for equations). The light-limited CO_2_ assimilation rate is:

19 $${A}_{j}=\mathrm{min}\left({A}_{j}^{ATP}, {A}_{j}^{NADPH}\right)$$

The model for the CO_2_ assimilation rate when the electron transport rate is not limiting (*A*_*c*_) is taken from Heckmann et al*.*^2^ and extended by a parameter representing the fraction of PSII activity in the bundle sheath cells, which affects O_2_ generation. This parameter is set to *p*. In the whole model, each limitation is considered independently; the plant’s CO_2_ assimilation rate is determined by the lower of the two limitations:

20 $$A =\mathrm{min}\left({A}_{j}, {A}_{c}\right)$$

### Temperature-dependence

Temperature affects the CO_2_ assimilation rate by changing the maximal activity of the C_4_ cycle, the carboxylation rate of Rubisco, and the electron transport rate. Temperature also affects the specificity of Rubisco and the Michaelis constants of Rubisco and PEPC. We model the temperature response by an extended Arrhenius function that describes two counteracting effects: rate increases with increasing temperature and enzyme inactivation through thermal instability^37^. We use parameters taken from literature or fitted to available data.

The extended Arrhenius function is given by Massad et al.^37^:

21 $$f\left(T\right)={k}_{25}\mathit{exp}\left[ E\frac{T- 298.15}{298.15 R T }\right]\frac{ \left[1 +\mathit{exp}\left(\frac{298.15 S - H}{298.15 R}\right)\right]}{\left[1+\mathit{exp}\left(\frac{T S -H}{T R}\right)\right]}$$

The parameters of the extended Arrhenius function are: (1) the value of the considered enzyme at temperatures 25 °C (*k*_*2*5_); (2) the activation energy (*E*); (3) the deactivation energy (*H*); (4) an entropy factor (*S*); (5) the universal gas constant (*R*); and (6) the temperature considered (*T*). (see Supplementary Methods S6 for details and Table S3 for the parameters).

### Data used in the analyses

As the raw data of Vogan and Sage^39^ were not available, we extracted it from the corresponding figures using the Graph Grabber software provided by Quintessa Limited (Version 1.5.5). The measured data include curves of the CO_2_ assimilation rate as a function of intercellular CO_2_ concentration (*C*_*i*_) and the ratio of atmospheric CO_2_ concentration (*C*_*a*_) and *C*_*i*_. We derive the CO_2_ concentration in the mesophyll cell (*C*_*m*_) for a given *C*_*a*_ by considering this *C*_*a*_/*C*_*i*_-ratio and assuming that the ratio of *C*_*m*_ to *C*_*i*_ is 0.85 (as CO_2_ enters the mesophyll through diffusion, the *C*_*m*_ / *C*_*i*_ ratio has to be below 1). As can be seen from our sensitivity analysis (see below and Supplementary Fig. S1), the exact value for the *C*_*m*_ / *C*_*i*_ ratio does not affect our conclusions.

To transform the in vitro PEPC activity given by Dwyer et al.^56^ to an in vivo activity, the in vitro value is divided by 3^57–59^.

### Required nitrogen re-allocation (δ_n_)

Required nitrogen re-allocation (*δ*_*n*_, [fraction]) is defined as the total fraction of nitrogen that needs to be re-allocated between photosynthetic pools to optimally adjust photosynthesis from the evolutionary scenario (${n}_{Etot}^{evo}$*, *${n}_{C4}^{evo}$*,* ${n}_{Jmax}^{evo}$) to a given experimental growth environment (${n}_{Etot}^{growth}$*, *${n}_{C4}^{growth}$*,* ${n}_{Jmax}^{growth}$):

22 $${\delta }_{n}={\sum }_{\mathrm{ i}\in \left\{Etot, C4, Jmax \right\}}\left|{n}_{i}^{evo}-{n}_{i}^{growth}\right|$$

### Statistical information

The differences between adaptation scenarios are tested with Wilcoxon rank sum tests. For details about the calculation of the resource allocation for the data set of Vogan and Sage^30^ (Fig. 3) see Supplementary Methods S8. All statistical analyses were conducted in R^52^. The difference of *δ*_*n*_ for various photosynthetic types was tested by sign tests.

## Results

### Optimal resource allocation in the evolutionarily relevant environment explains physiological data and outperforms models based on the experimental growth environment in C_4_*Flaveria* plants

Do photosynthetic types exhibit differences in phenotypic plasticity, i.e., do they differ in their ability to adjust their photosynthetic resource allocation to optimally fit the environment in which they were grown? Or is resource investment static and reflects past environments in which the plants’ ancestors evolved? To compare these competing hypotheses in the genus *Flaveria*, we predict physiological data of plants that are either optimally adapted to the experimental growth conditions (EGC) used in the respective studies (‘growth scenario’) or to the environments in which they likely evolved (‘evolutionary scenario’); with respect to our model, these environments differ in terms of atmospheric CO_2_ concentration, temperature, and light intensity (Supplementary Tables S4-S7). This in silico experiment also serves as validation for our modeling framework; if the parameterization for *Flaveria* and our optimality assumptions are correct, we would expect the model to explain physiological responses in one of the two or in an intermediate scenario.

To predict the physiological data of plants that are optimally adapted to the evolutionary scenario, we use our model to identify the optimal resource allocation for C_3_, C_3_–C_4_ intermediate, and C_4_ *Flaveria* species in the evolutionary environment. This environment is based on the suggested environment of C_4_ evolution in *Flaveria*^13,19,20^, with high light intensities, high temperature, and 280 µbar atmospheric CO_2_ concentration (see Supplementary Table S4 for parametrization). For comparison, we identify the optimal resource allocation under the EGCs of the following studies, which provide information about all considered environmental factors: (1) Vogan and Sage^39^, (2) Vogan and Sage^30^, and (3) Dwyer et al*.*^56^. Vogan and Sage^39^ measured the net CO_2_ assimilation rate as a function of intercellular CO_2_ concentration (A-C_i_ curve) and as a function of temperatures between 15 °C and 45 °C for C_3_, C_3_–C_4_ intermediate, and C_4_ *Flaveria* species. In this experiment, plants were grown at light intensities of 560 µmol quanta m^−2^ s^−1^, 37 °C at daytime, current atmospheric O_2_ concentration, and 380 µbar or 180 µbar atmospheric CO_2_ concentrations (Supplementary Table S5). In an independent experiment, Vogan and Sage^30^ measured the dependence of CO_2_ assimilation rate on leaf nitrogen levels in C_3_, C_3_–C_4_ intermediate, C_4_-like, and C_4_ *Flaveria* species. The plants were grown at 554 µmol quanta m^−2^ s^−1^ light intensity, 30 °C at daytime, at 380 µbar atmospheric CO_2_ and current atmospheric O_2_ concentrations (Supplementary Table S6). Dwyer et al*.*^56^ performed detailed experiments on the photosynthetic resource allocation and performance of the C_4_ species *F. bidentis*. The Dwyer et al*.*^56^ data set allows us to compare the predicted nitrogen investment into the three major photosynthetic components—Rubisco, C_4_ cycle, and electron transport chain—, and the corresponding CO_2_ assimilation rate, to experimentally observed resource allocation patterns. The plants were grown under 25 °C or 35 °C at daytime, 550 µmol quanta m^−2^ s^−1^, 380 µbar CO_2_, and current atmospheric O_2_ concentrations (Supplementary Table S7).

In the three studies, the experimental measurement conditions (EMC) differ from both the EGC and the evolutionary condition. Typically, the EMC shows higher light intensities than the EGC. In contrast, the major difference between the evolutionary environment and the EMC is the atmospheric CO_2_ concentration. There are additional differences between the conditions that are study-specific, e.g., differences in temperature; detailed comparisons of conditions are listed in Supplementary Tables S4–S7.

**Figure 2:**
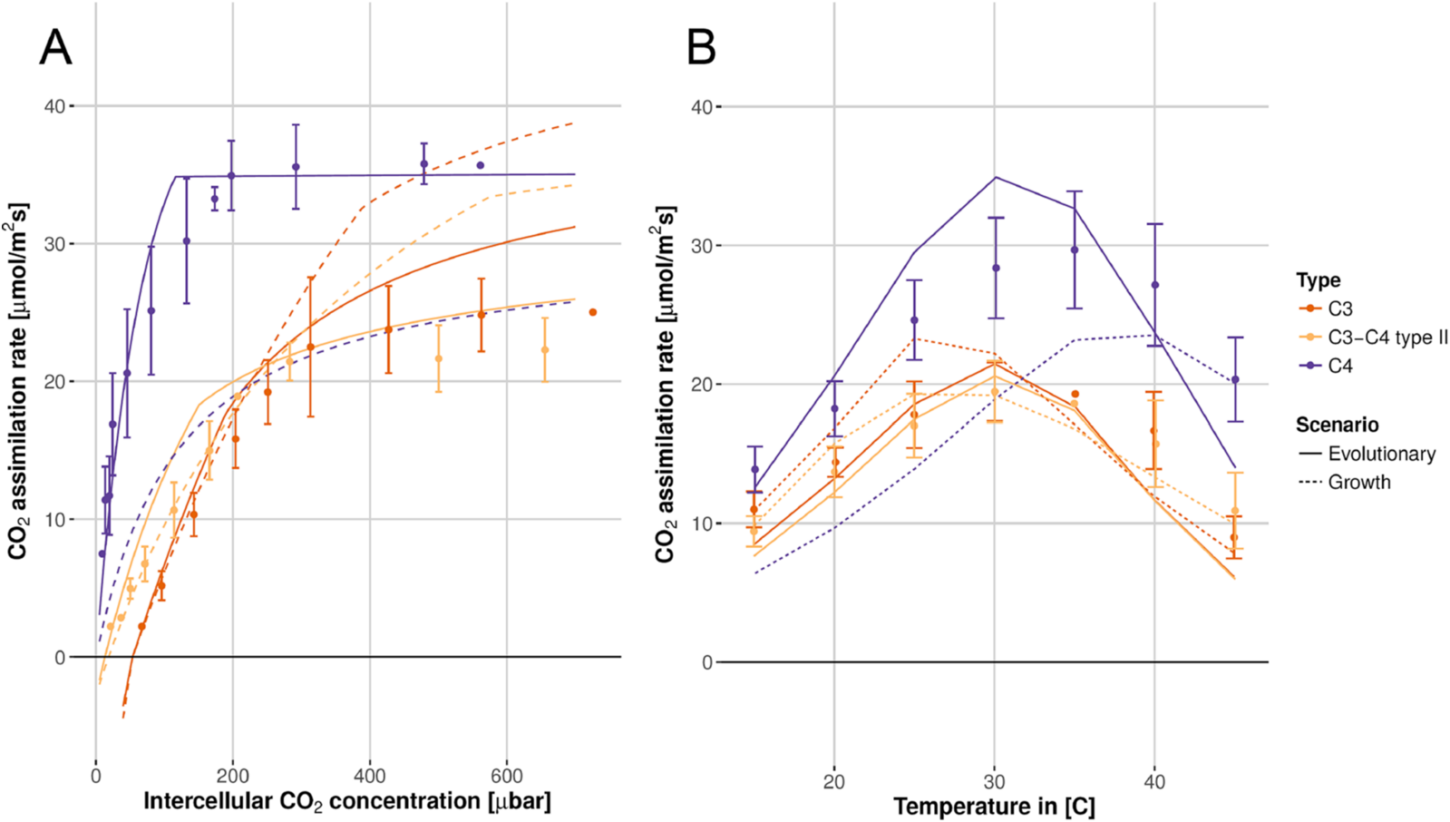
Model results based on optimality in the evolutionary scenario (solid lines) describe the measured data (circles ± SE) better than the model assuming optimal adaptation to the EGC (dashed lines) for *F. robusta* (C_3_), *F. ramosissima* (C_3_–C_4_), and *F. bidentis* (C_4_) grown at the 380 µbar atmospheric CO_2_ (data from Vogan and Sage^39^). (**A**) The net CO_2_ assimilation rate as a function of intercellular CO_2_ concentration, measured at 30 °C. SE was calculated based on three independently measured plants. (**B**) The net CO_2_ assimilation rate as a function of temperature at 380 µbar atmospheric CO_2_ concentration. See Supplementary Table S9 for the residual sum of squares. Figure created using R 4.0^52^.

**Figure 3:**
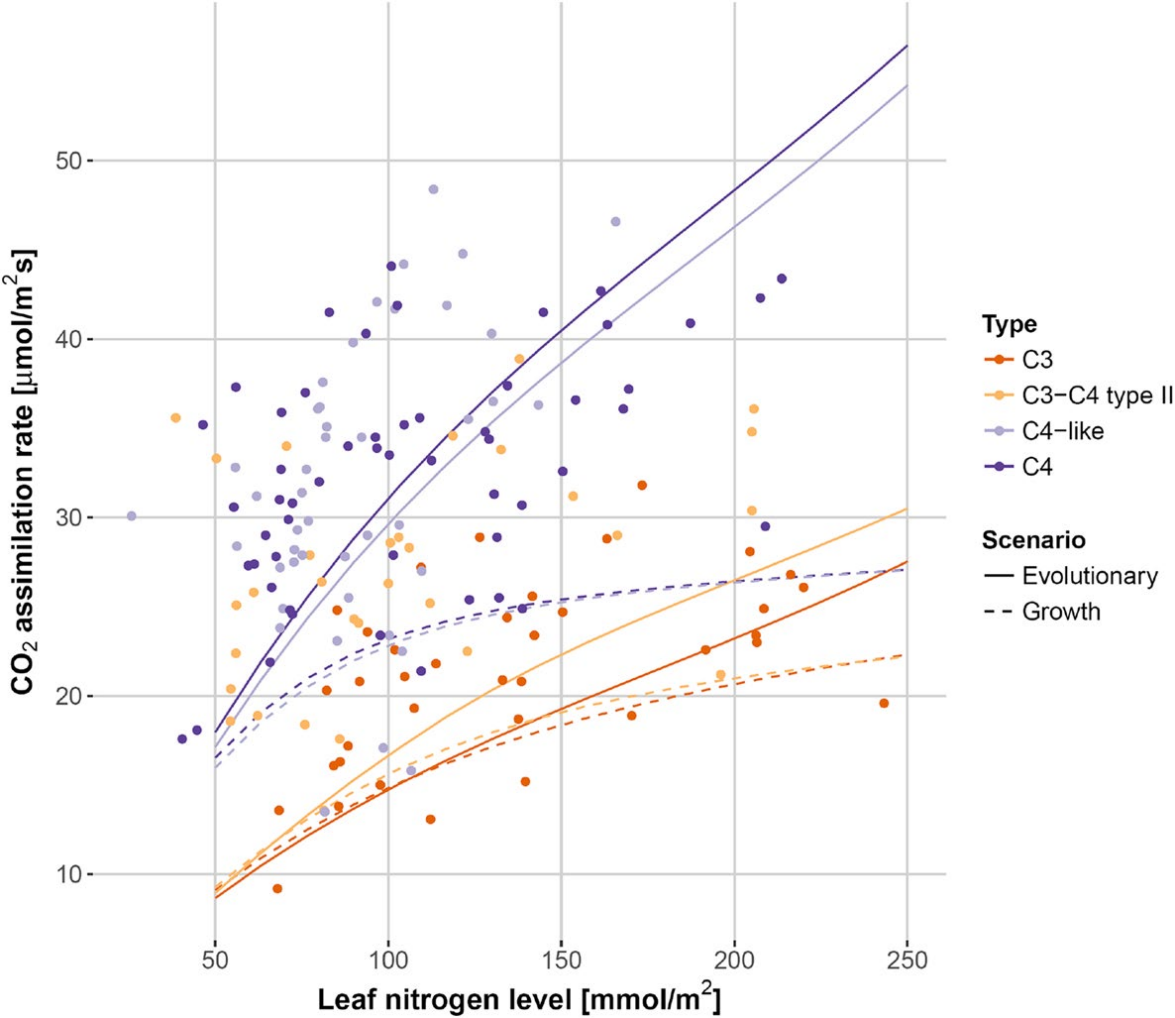
The dependence of the CO_2_ assimilation rate on leaf nitrogen levels for various *Flaveria* species is consistent with model results based on optimality in the evolutionary scenario (solid lines). For C_3_-C_4_ intermediate, C_4_-like, and C_4_, these results outperform results from simulations assuming optimal phenotypic adaptation to the EGC (dashed lines). The modeled species are *F. pringlei* (C_3_)*, F. floridana* (C_3_-C_4_)*, F.* *palmeri* (C_4_-like)*, and F. bidentis* (C_4_) (data from Vogan and Sage^30^). See Supplementary Table S9 for the residual sum of squares. Figure created using R 4.0^52^.

For C_3_ *Flaveria* species (*F. pringlei* or *F. robusta*), the model results assuming an optimal allocation under the evolutionary scenario agree qualitatively with the measured data of Vogan and Sage^30,39^, and visually, they appear to fit the data better than results assuming optimality under the EGC^30^ (Figs. 2, 3; Supplementary Figs. S2–S5). To allow a statistical comparison between the quality of the two predictions, for each of the two scenarios, we calculated the squared residuals across all C_3_ *Flaveria* data points in Figs. 2, 3 and Supplementary Figs S2–S5 (see Supplementary Table S9); these two distributions were then compared through a Wilcoxon rank sum test. This test was not statistically significant at the 5% level (*P* = 0.31). Thus, it is possible that the somewhat better fit for the evolutionary scenario is caused by random fluctuations or experimental errors rather than by a superiority of one scenario over the other.

The result was very similar for the C_3_-C_4_ intermediates, *F. ramosissima* and *F. floridana.* Again, the predictions assuming an optimal allocation under the evolutionary scenario agree qualitatively with the measured data of Vogan and Sage^30,39^, and seem to fit the data better than predictions under the EGC (Figs. 2, 3; Supplementary Figs. S2–S5); however, the prediction errors are again not statistically significantly different between the two scenarios (*P* = 0.86, Wilcoxon rank sum tests, Supplementary Table S9).

The C_4_-like species *F. palmeri* is only considered in the data set of Vogan and Sage^30^ (Fig. 3). The model results for *F. palmeri* assuming optimal resource allocation in the evolutionary scenario are consistent with the measured data. The squared residuals for the evolutionary scenario is significantly smaller than that for the growth scenario (*P* = 0.02, Wilcoxon rank sum tests, Supplementary Table S9).

Focusing on the C_4_ species *F. bidentis*, curves calculated from a model parameterized for optimal CO_2_ assimilation in the EGC are qualitatively different from the experimental curves of Vogan and Sage^30,39^ (Figs. 2, 3; Supplementary Figs. S2–S5), except for the *A*-*C*_*i*_ curves measured at low CO_2_ levels, for both 30 °C and 40 °C. In contrast, the modeled curves based on a model optimally adapted to the evolutionary scenario are qualitatively consistent with the measured curves. Jointly considering all measured curves in Figs. 2, 3 and Supplementary Figs. S2–S5^30,39^, we find that the squared residuals for the evolutionary scenario is statistically significantly smaller than that for the growth scenario (*P* = 8.3 × 10^–5^, Wilcoxon rank sum tests, Supplementary Table S9).

**Figure 4:**
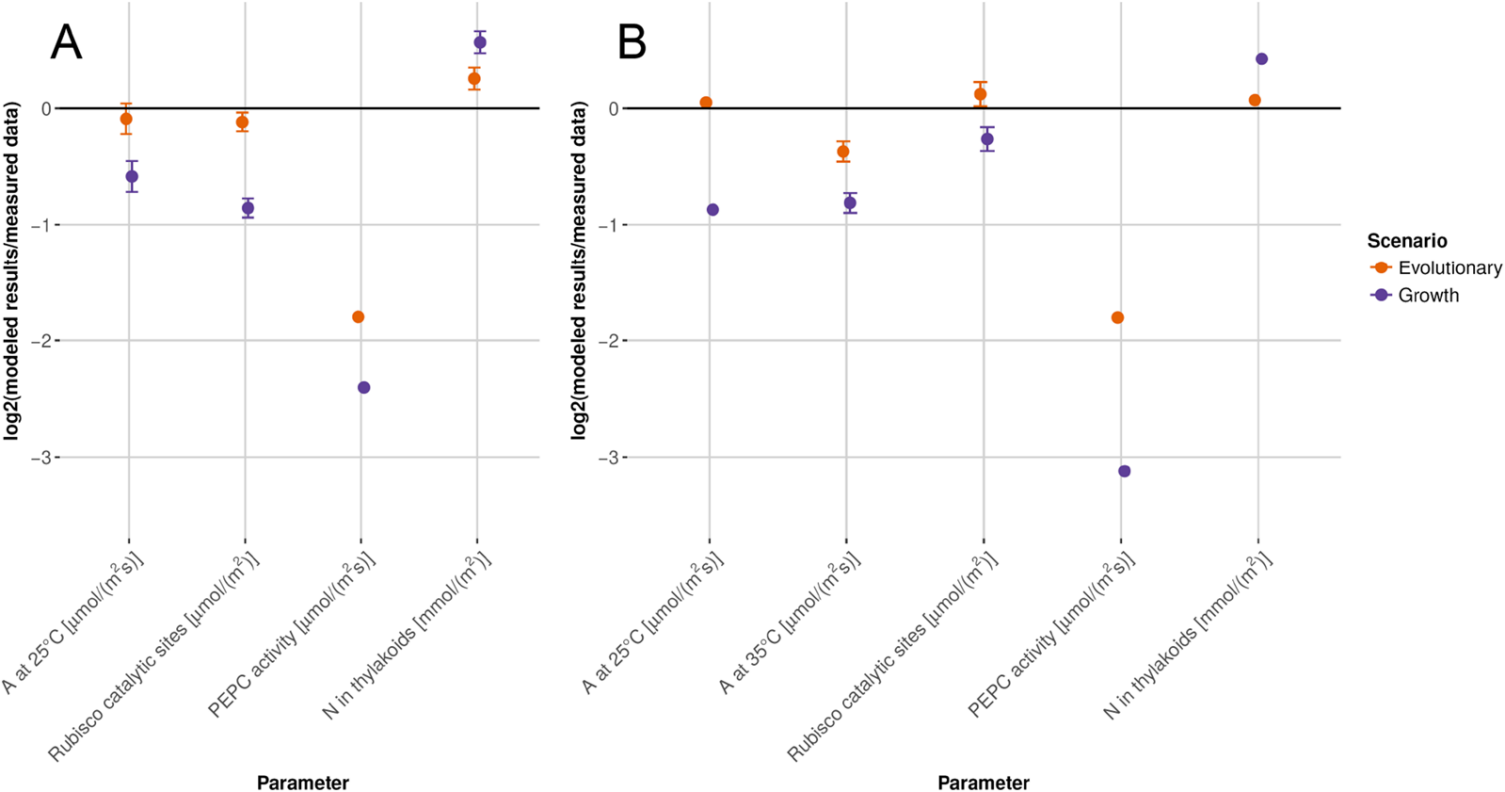
A detailed analysis of resource allocation and physiology in *F. bidentis* (C_4_) shows a good agreement between experimental data^56^ and model results based on the evolutionary scenario (orange circles). Alternative model results assuming optimal phenotypic adaptation to the EGC consistently show higher disagreement with the data (purple circles). Values are mean log_2_(modeled results/measured data) ± SE. (**A**) Plants grown at 25 °C (**B**) Plants grown at 35 °C. *A* = net CO_2_ assimilation rate; *N* = nitrogen. See Dwyer, et al.^56^ for sample sizes. Figure created using R 4.0^52^.

Dwyer et al*.*^56^ performed detailed experiments on the photosynthetic resource allocation and performance of the C_4_ species *F. bidentis*. First, we analyze the discrepancy of each model prediction with the empirical measurement. Model predictions of chlorophyll content and the amount of photosystem II agree within a factor of 1.10 to 1.22 (this corresponds to a factor 0.13 to 0.28 assuming a log2-scale as presented in Fig. 4) with values measured by Dwyer et al.^56^ (see Supplementary Table S10 for absolute values). For plants grown at 25 °C, the resource allocation determined under the evolutionary scenario agrees with the measured data within a factor of 0.29 to 1.19 (this corresponds to a factor of − 1.8 to 0.25 assuming a log2-scale; Fig. 4A); at 35 °C, agreement is within a factor of 0.29 to 1.09 (this corresponds to a factor − 1.8 to 0.12 assuming a log2-scale; Fig. 4B). In both cases, agreement is much lower for predictions in the growth scenario (which are 0.10 to 1.42 or − 3.26 to 0.50 on a log2-scale for 25 °C (Fig. 4A) and 0.11 to 1.34 or − 3.12 to 0.42 on a log2-scale for 35 °C (Fig. 4B)). Then, we analyze the overall discrepancy of model prediction and empirical measurement presented in Fig. 4. We determine the deviation (‘error’) between all model predictions and measurements as the squared residuals (normalized to fractions of the experimental means).We assessed the statistical significance of the superior performance of the evolutionary scenario (compared to the growth scenario) by comparing the errors. The resource allocation calculated for the evolutionary scenario outperforms the growth scenario for the data represented in Fig. 4 (*P* = 1.0 × 10^–4^, Wilcoxon rank sum test). In Fig. 4, there is a discrepancy between measured in vitro PEPC activity and predicted in vivo activity, a disparity that has been noted before^57–59^. When in vitro PEPC activity is corrected using independent data on in vitro-in vivo differences (Supplementary Fig. S8; for derivation see Methods), the model successfully predicts all measurements; the agreement is within a factor of 0.86 to 1.19 at 25 °C and 0.77 to 1.09 at 35 °C (this corresponds to a factor of − 0.21 to 0.25 at 25 °C and − 0.37 to 0.12 at 35 °C assuming a log2-scale).

Although we could obtain the majority of our model parameters from the literature, the relationship of cytochrome f and the maximal electron transport rate of the CET had to be estimated (see Methods). We performed a sensitivity analysis to examine the robustness of the results to changes in the estimated parameters and to uncertainties in values obtained from the literature, focusing on parameters with high uncertainty or major expected effect on model predictions (Supplementary Methods S7 and Table S11). The predictions based on the evolutionary scenario outperform those based on the growth environment consistently across all parameter sets (Supplementary Fig. S1).

Adjustments in the nitrogen allocation require substantial changes to protein abundances, which can only be achieved through massive protein breakdown and de-novo synthesis (see Moejes, et al.^60^ for a general discussion and Schmollinger, et al.^61^ for an example in *Chlamydomonas*). Thus, we assume that plants require multiple hours to days in order to adjust their protein levels to a new environmental condition. Accordingly, we assume that plants cannot adapt their resource allocation patterns on the timescale of a measurement, which lasts on the order of minutes to hours. This is our rationale for simulating plants optimally adapted to the EGC, even when analyzing data collected at rather different EMCs. However, it is conceivable that at least the energy allocation, including the proportion of LET, can adjust to the EMC on the timescale of the experiment. We thus performed simulations under an alternative model, where nitrogen allocation is optimized for the EGC, but energy allocation is subsequently optimized for the EMC. The results are qualitatively similar to the above results from simulations where both nitrogen and energy allocation are optimized for the EGC (Supplementary Figs. S9–S16).

### The model suggests a unique evolutionary environment for C_4_ photosynthesis in *Flaveria*

**Figure 5:**
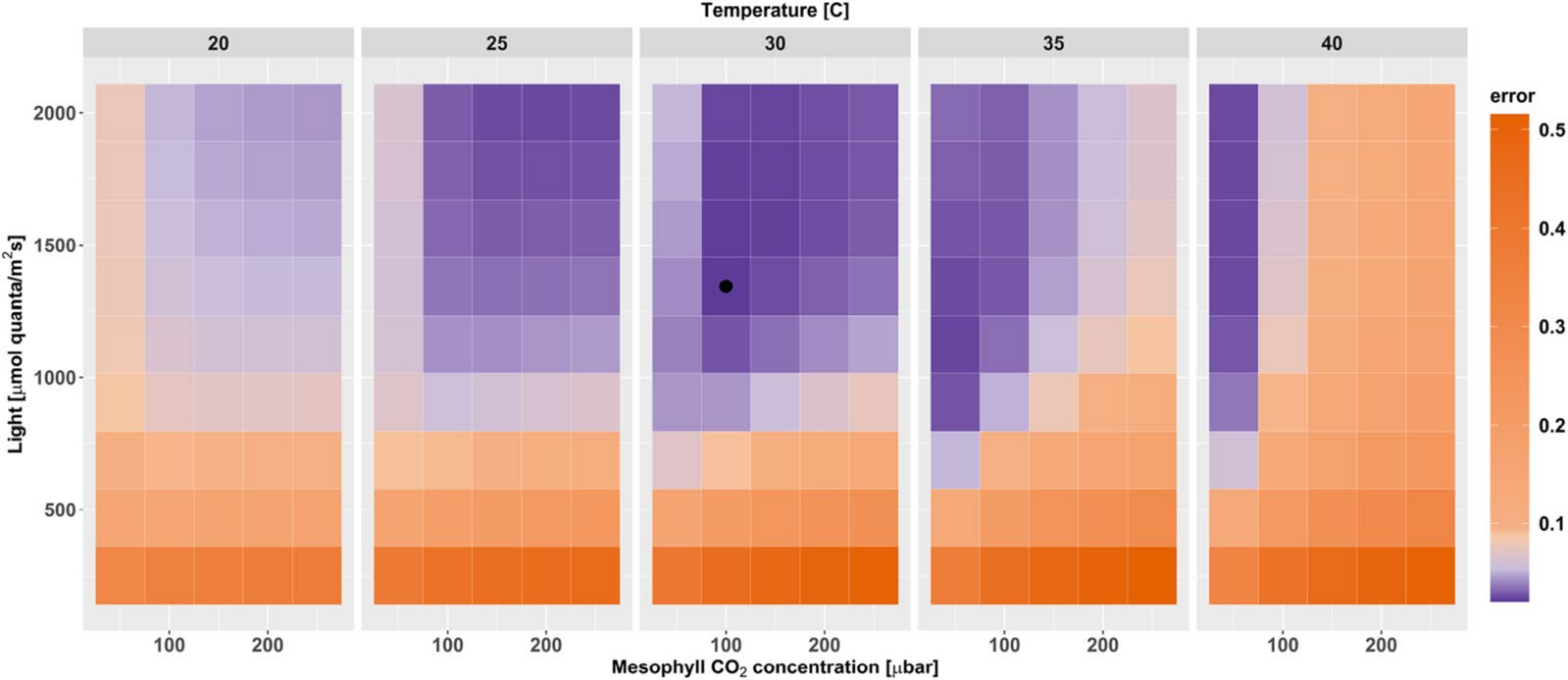
Discrepancy between measured and modeled *F. bidentis* data across diverse environments. The black circle indicates the environment that best explains the experimental data of Dwyer, et al.^56^. The deviation between model predictions and measurements (‘error’) is defined as the mean of the squared residuals (which are expressed as fractions of experimental means). Figure created using R 4.0^52^.

Compared to a parameterization optimized for the growth scenario, the model optimally adapted to the evolutionary scenario leads to superior predictions of plant performance and resource allocation in C_4_ plants across diverse physiological data sets. The inferior performance of the growth scenario model indicates a lack of phenotypic plasticity of resource allocation in C_4_ plants, a result that is in agreement with previous reports based on experimental observations^15^. The lack of phenotypic plasticity points to the possibility that the environment most relevant for recent evolutionary adaptation of a given C_4_ plant could be inferred quantitatively from observations on plant physiology and resource allocation. Thus, to infer a typical evolutionary environment for C_4_ *Flaveria bidentis,* we calculated optimal resource allocation under conditions covering plausible ranges of mesophyll CO_2_ partial pressure, temperature, and light intensities, and we then identified the conditions that best explain the empirical data (Fig. 5). As atmospheric O_2_ concentration remained almost constant for at least the last few million years^23^, this environmental parameter is set to a constant value. We compare the simulations to the empirical data of Dwyer et al.^56^, as this data set comprises detailed measurements for each nitrogen pool and the resulting CO_2_ assimilation rate, allowing us to quantify the discrepancy between modeled and measured values as the mean squared residuals (normalized to fractions of experimental means).

The model environment that shows the smallest prediction error defines a unique environment (Fig. 5), characterized by 1343.75 µmol quanta m^−2^ s^−1^ light intensity, 30 °C, a mesophyll CO_2_ level of 100 µbar, and an O_2_ level of 200 mbar. This environment corresponds to an atmospheric CO_2_ concentration of about 280 µbar (Supplementary Table S8). Some similar environments lead to only slightly worse fits to the empirical data; the areas in which the model successfully describes the empirical values generally show high light intensities, intermediate to high temperatures, and a trend towards low CO_2_ partial pressures (Fig. 5).

In contrast to our findings for C_4_ and C_4_-like plants, the performance of the evolutionary and the growth scenario models is similar for C_3_ and C_3_–C_4_ intermediate *Flaveria* species (Figs. 2, 3; Supplementary Figs. S2–S5 and Table S9). It is conceivable that the lack of superior performance for the evolutionary scenario in C_3_ *Flaveria* species is due to an inappropriate parameterization of the evolutionary scenario. The environment most relevant for the recent evolution of C_3_ *Flaveria* may be different from the environment used in the simulations, which was chosen based on its relevance for the C_4_ lineages. To explore this possibility, we simulated a wide range of alternative environments, testing if resource allocation optimized for any of these leads to significantly improved model predictions for the data from Vogan and Sage^39^ for C_3_ plants. However, none of the environments tested led to a significant improvement (Supplementary Figs. S6–S7).

Optimal resource allocation patterns are determined by an interplay between the different environmental factors. For C_4_ species, high light intensities (as in the evolutionary scenario) tend to favor an increased nitrogen investment into the dark reactions, which goes along with a reduced investment into the electron transport chain. The effect of temperature is of special importance for plants using the C_4_ cycle, as temperature increases PEPC activity drastically^37^ and therefore reduces the necessary nitrogen investment into the C_4_ cycle. This allows an increased investment into Rubisco and the electron transport chain, both of which show reduced activity at elevated temperatures due to thermal instabilities. Lower mesophyll CO_2_ levels tend to increase the investment into the C_4_ cycle while decreasing the investment into the electron transport chain and (albeit by a small factor) into Rubisco.

### Limited phenotypic plasticity is linked to a high requirement of nitrogen re-allocation

Our results indicate that C_4_ *Flaveria* species show a lower degree of photosynthetic phenotypic plasticity than closely related C_3_ species (indicated by the inferior performance of the growth model compared to the evolutionary scenario for C_4_ *Flaveria* species, there is no significant difference observed in C_3_ *Flaveria* species; Figs. 2, 3 and Supplementary Figs S2–S5). On a molecular level, phenotypic plasticity predominantly requires the re-allocation of nitrogen between the major photosynthetic protein pools, in addition to post-translational control. After finding the optimal nitrogen allocation patterns in the evolutionary and growth scenarios, we calculated the absolute difference in the fraction of photosynthetic nitrogen allocated to each major pool of photosynthetic nitrogen (Calvin-Benson cycle; C_4_ cycle; electron transport). We then summed these fractions to quantify the total fraction of nitrogen that needs to be re-allocated between photosynthetic pools to adjust photosynthesis between the two optimal nitrogen allocation patterns (*δ*_*n*_, see Methods). Table 2 shows this amount of nitrogen re-allocation for C_3_, C_3_-C_4_ intermediate, C_4_-like, and C_4_ *Flaveria* species at four different leaf nitrogen levels. We find that photosynthetic types that utilize C_4_ photosynthesis require a consistently higher amount of re-allocation compared to C_3_ plants (*P* = 1.5 × 10^–5^, sign test). Our results thus indicate a link between required nitrogen re-allocation and limited photosynthetic phenotypic plasticity, suggesting a possible causal relationship.

**Table 2:** Required nitrogen re-allocation (*δ*_*n*_, [fraction]) for different leaf nitrogen levels for various *Flaveria* species.

	Leaf nitrogen level			
	50 mmol m^−2^	130 mmol m^−2^	170 mmol m^−2^	250 mmol m^−2^
*F. pringlei* (C_3_)	0.039	0.105	0.151	0.273
*F. floridana* (C_3_-C_4_)	0.072	0.159	0.222	0.360
*F. palmeri* (C_4_-like)	0.100	0.263	0.325	0.415
*F. bidentis* (C_4_)	0.109	0.275	0.334	0.414

The required nitrogen re-allocation represents the total fraction of nitrogen that needs to be re-allocated between photosynthetic pools to optimally adjust photosynthesis from the evolutionary scenario to a given experimental growth environment.

## Discussion

Our novel modeling framework allows us to study the interplay between photosynthetic performance, the environment, and resource investment on the molecular level. Comparisons of model predictions with phenotypic and molecular data from the genus *Flaveria* (Figs. 2, 3, 4) show that models of C_4_ plants adapted to an evolutionary environment outperform models that consider the experimental growth conditions. These results suggest a low phenotypic plasticity in terms of resource allocation in C_4_ plants of the model genus *Flaveria*, supporting earlier hypotheses on a low photosynthetic plasticity of C_4_ plants^15^. In a recent study, Pignon and Long^62^ found that C_4_ plants do not appear to have adapted their photosynthetic gene expression to modern levels of atmospheric CO_2_, a result that confirms a low phenotypic plasticity in these plants. This limited phenotypic plasticity may potentially be explained by the large amount of nitrogen that needs to be re-allocated by C_4_ plants to optimally adapt to a given growth environment (Table 2): adaptation of C_4_ photosynthesis requires more drastic changes in gene expression than C_3_ photosynthesis. The relatively young age of many C_4_ species compared to their C_3_ ancestors^63^ might further enhance this effect, because the required gene-regulatory networks had less time to evolve than those of their C_3_ ancestors. Plants with low photosynthetic phenotypic plasticity might contain information about their adaptive environment in their relatively static gene expression patterns. Based on this reasoning, we make quantitative predictions for the environments that dominated the recent evolution of C_4_ *Flaveria* (Fig. 5). Previously, environments relevant for C_4_ photosynthesis evolution have been inferred—mostly qualitatively—based on C_3_-C_4_ habitat comparisons^13,19,20^ and geophysiological considerations^21^. Our results are consistent with and refine these earlier estimates.

When C_4_ species grow under low CO_2_ levels, the model assuming optimality in the growth scenario explains the measured data better than the evolutionary model (Supplementary Figs. S3–S4 and Table S9). To some extent this is consistent with the results presented in Fig. 5, where lower mesophyll CO_2_ concentrations and light intensity than assumed in the evolutionary scenario lead to a better fit of simulated and measured data, thus refining our prior assumptions about the evolutionary environment.

Although the predictions for total nitrogen investment into the thylakoids based on the evolutionary scenario are highly consistent with the measurements performed by Dwyer et al.^56^, the model overestimates the amount of cytochrome f by a factor of 2 (1.56 µmol m^−2^ instead of the measured 0.87 µmol m^−2^ for plants grown at 25 °C, 1.35 µmol m^−2^ instead of 0.80 µmol m^−2^ at 35 °C, see Supplementary Table S10). However, the experimental error of the measurements is uncertain, as no replicate measurements were performed for this parameter^56^. Discrepancies between model predictions and observations may also be in part due to error propagation from modeled amounts of chlorophyll and the photosystems. In each simulation, we optimized resource allocation for an environment that represents a static approximation to the dynamic environment a plant is facing. As diurnal and annual variations (which are no focus of this work) potentially show short-term trade-offs^44,64^, these might lead to a discrepancy between modeled and real evolutionary scenarios. In particular, the natural ancestral habitat must have exhibited periodically as well as randomly fluctuating conditions, compared to the stable EGCs in audited growth chambers and the statically modeled evolutionary scenario.

Given the complexity of our physiological model, we needed to make a number of assumptions. We addressed uncertainties in model parameters through sensitivity analyses, showing that our conclusions are robust against variation in these parameters (Supplementary Fig. S1). Furthermore, our predictions assume that nitrogen availability in the evolutionary scenario was identical to current nitrogen availability. As the role of nitrogen availability in C_4_ evolution remains unclear, further research is needed to assess the effect of nitrogen availability on plants under the ancestral, current, and transitional environments. Furthermore, while our approach of maximizing the assimilation rate per available CO_2_ concentration will account for water-use efficiency implicitly, a promising avenue for future evolutionary studies will be the explicit inclusion of stomatal responses (see, e.g., Bellasio and Farquhar^65^).

There are only a limited number of data sets available that include the information for each considered environmental factor. In the three available data sets that included all necessary information, plants were not grown under the same conditions under which experiments were performed (i.e., EGC and EMC differed). The EGC and EMC show their biggest difference in the light intensities, but other factors differ also, e.g., temperature (Supplementary Tables S4–S7). While the disparity between EGC and EMC complicated the analysis and interpretation, we argue that the analysis of different photosynthetic types (C_3_, C_3_–C_4_ intermediates, and C_4_) across a wide range of environmental conditions provides a solid basis for the presented results. The complexity of the analysis is reduced by considering the model genus *Flaveria* that allows us to focus on the effect of different photosynthetic types rather than differences across genera.

In contrast to the findings in C_4_ and C_4_-like plants, the predictive performance of the evolutionary and the growth scenario models is similar for C_3_ and C_3_–C_4_ intermediate *Flaveria* species (Supplementary Table S9). This similarity could be caused by the similar assimilation rates found for the evolutionary and growth scenario models in C_3_ and C_3_–C_4_ plants, which make it difficult to quantify model performance on noisy data (Figs. 2, 3 and Supplementary Figs. S2–S5). Overall, our results point to a higher phenotypic plasticity of C_3_ and C_3_–C_4_ intermediate plants compared to C_4_ and C_4_-like plants. Thus, in contrast to the latter photosynthetic types, it may not be possible to estimate ancestral evolutionary environments for C_3_ plants based on our approach.

Our model provides a powerful tool to analyze the resource allocation of photosynthetic organisms and its dependence on environmental factors, allowing estimates for the maximal electron transport rate for LET and CET, the proportion of LET and CET as well as the nitrogen and energy allocation for which measurements are currently infeasible or impractical. This may prove to be of particular utility for systematically assessing the likely performance of crops in environments distinct from their natural habitats and for suggesting engineering targets in cases of limited phenotypic plasticity.

## Supplementary Information

Supplementary Information.
