# Supplementary material for: Modeling photosynthetic resource allocation connects physiology with evolutionary environments
Source: Sci Rep. 2021 Aug 5;11:15979. doi: 10.1038/s41598-021-94903-0 (PMC8342476; doi:10.1038/s41598-021-94903-0)
Supplement: Supplementary file 1 — Supplementary Information. [file 41598_2021_94903_MOESM1_ESM.pdf]

Supplementary Information

Modeling Photosynthetic Resource Allocation Connects Physiology with Evolutionary Environments

Esther M. Sundermann, Martin J. Lercher, David Heckmann

List of Supplementary Items

Supplementary Figures p.3

Fig. S1 Sensitivity analysis.

Fig. S2 A-C_i curve measured at 40°C using plants grown at 380 µbar atmospheric CO_2.

Fig. S3 A-C_i curve measured at 30°C using plants grown at 180 µbar atmospheric CO_2.

Fig. S4 A-C_i curve measured at 40°C using plants grown at 180 µbar atmospheric CO_2.

Fig. S5 A-Temperature curve using plants grown at 180 µbar atmospheric CO_2.

Fig. S6 Discrepancy between measured and modeled results of *F. robusta* (C_3) across diverse environments assuming no phosphate-limitation.

Fig. S7 Discrepancy between measured and modeled results of *F. robusta* (C_3) across diverse environments assuming phosphate-limitation.

Fig. S8 A detailed analysis of resource allocation and physiology in *F. bidentis* (C_4), considering *in vivo* PEPC activity.

Fig. S9 A detailed analysis of resource allocation and physiology in *F. bidentis* (C_4), assuming an optimal energy allocation under the experimental measurement conditions.

Fig. S10 A-C_i curve measured at 30°C using plants grown at 380 µbar atmospheric CO_2 for various *Flaveria* species, assuming an optimal energy allocation under the experimental measurement conditions.

Fig. S11 A-C_i curve measured at 30°C using plants grown at 180 µbar atmospheric CO_2 for various *Flaveria* species, assuming an optimal energy allocation under the experimental measurement conditions.

Fig. S12 A-C_i curve measured at 40°C using plants grown at 380 µbar atmospheric CO_2 for various *Flaveria* species, assuming an optimal energy allocation under the experimental measurement conditions.

Fig. S13 A-C_i curve measured at 40°C using plants grown at 180 µbar atmospheric CO_2 for various *Flaveria* species, assuming an optimal energy allocation under the experimental measurement conditions.

Fig. S14 A-Temperature curve using plants grown at 380 µbar atmospheric CO_2 for various *Flaveria* species, assuming an optimal energy allocation under the experimental measurement conditions.

Fig. S15 A-Temperature curve using plants grown at 180 µbar atmospheric CO_2 for various *Flaveria* species, assuming an optimal energy allocation under the experimental measurement conditions.

Fig. S16 The dependence of the CO_2 assimilation rate on leaf nitrogen levels for various *Flaveria* species, assuming an optimal energy allocation under the experimental measurement conditions.

Supplementary Tables **p.17**

Table S1 *Flaveria* parametrization.

Table S2 Lower and upper bounds for the model parameters subject to numerical optimization.

Table S3 The parameters of the temperature-dependent model.

Table S4 The parametrization of the evolutionary environment.

Table S5 The parametrization of the experimental measurement and experimental growth environments of the data set from Ref. 1.

Table S6 The parametrization of the experimental measurement and experimental growth environments of the data set from Ref. 2.

Table S7 The parametrization of the experimental measurement and experimental growth environments of Ref. 3.

Table S8 Mesophyll CO_2 concentration as a function of atmospheric CO_2 concentration and photosynthetic type.

Table S9 In C_4 and C_4-like plants, the evolutionary scenario shows significantly smaller residual sum of squares compared to the growth scenario.

Table S10 The modeled and measured data of chlorophyll and PSII of *F. bidentis* (C_4).

Table S11 Distribution parameters used to generate the random parameter sets for the sensitivity.

Supplementary Methods **p.30**

Supplementary Figures

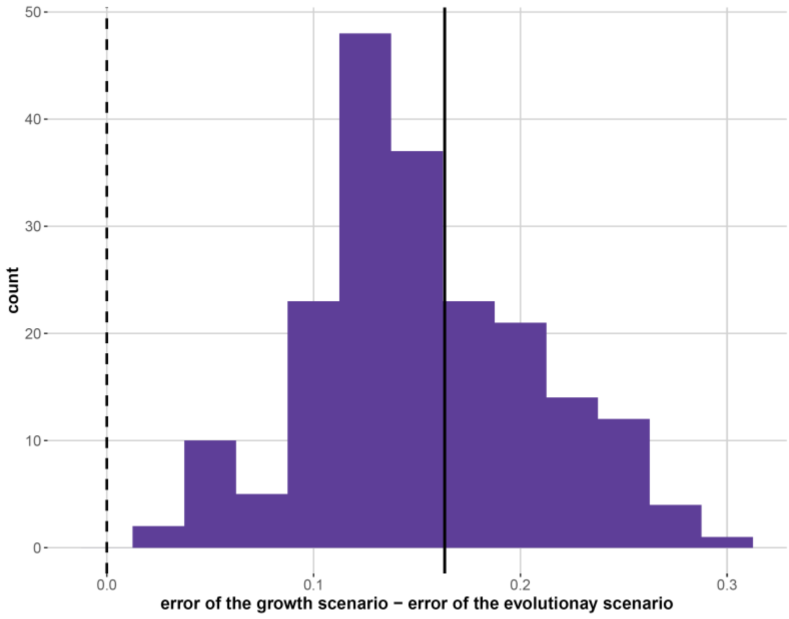

Fig. S1: Sensitivity analysis for our finding that for the C_4 plant *F. bidentis,* all predictions based on the evolutionary scenario outperform those based on the experimental growth environment. Related to Figures 2-4. We randomly perturbed uncertain model parameters to sample their effect on predictive performance of allocation data taken from Dwyer et al.[3] (see Methods S8 and Table S11 for details). The histogram shows the difference between the prediction error assuming an optimal resource allocation under the evolutionary scenario and under the experimental growth scenario, calculated for 200 randomly chosen sets of parameters. The error describes the mean squared residuals (expressed as fractions of the experimental means) for the data shown in Fig. 4a. The solid line represents the difference for the standard parametrization as shown in Fig. 4a and the dashed line represents the zero intercept. Figure created using R 4.0 (https://www.R-project.org/).

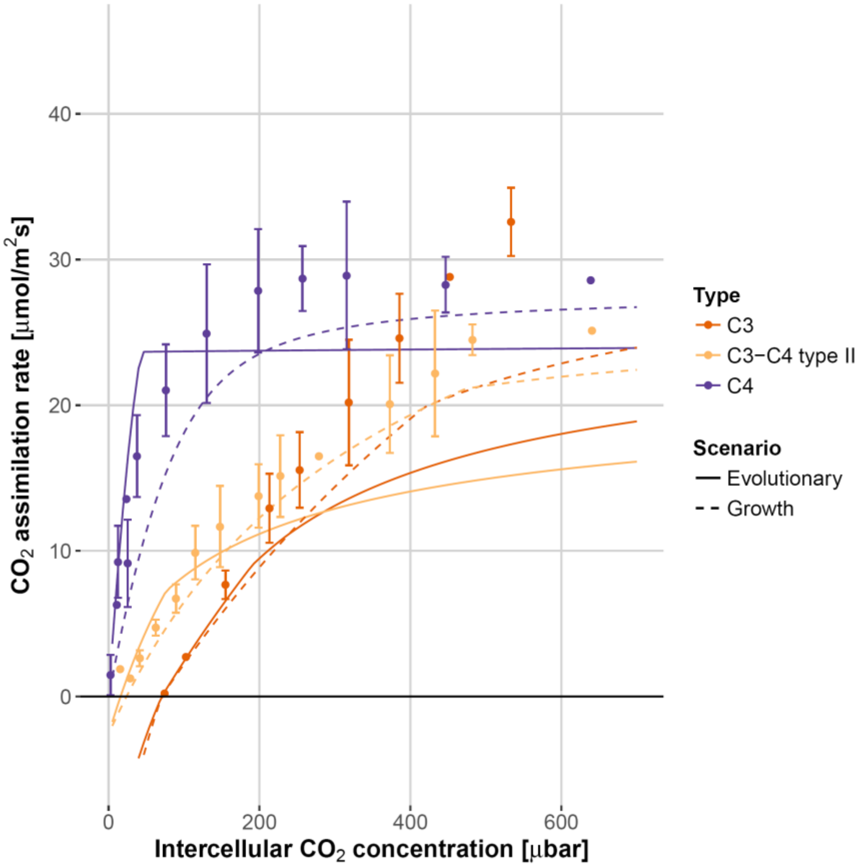

Fig. S2: Modeled results based on the evolutionary scenario (solid lines) describe the measured data (dots ± SE) better than the model assuming optimal adaptation to the experimental growth conditions (dashed lines) for *F. bidentis* (C_4). Related to Fig. 2a, but the A-C_i curve was measured at 40°C (data from Vogan and Sage[1]). Missing error bars result from unknown empirical errors. See Table S9 for the residual sum of squares. Figure created using R 4.0 (https://www.R-project.org/).

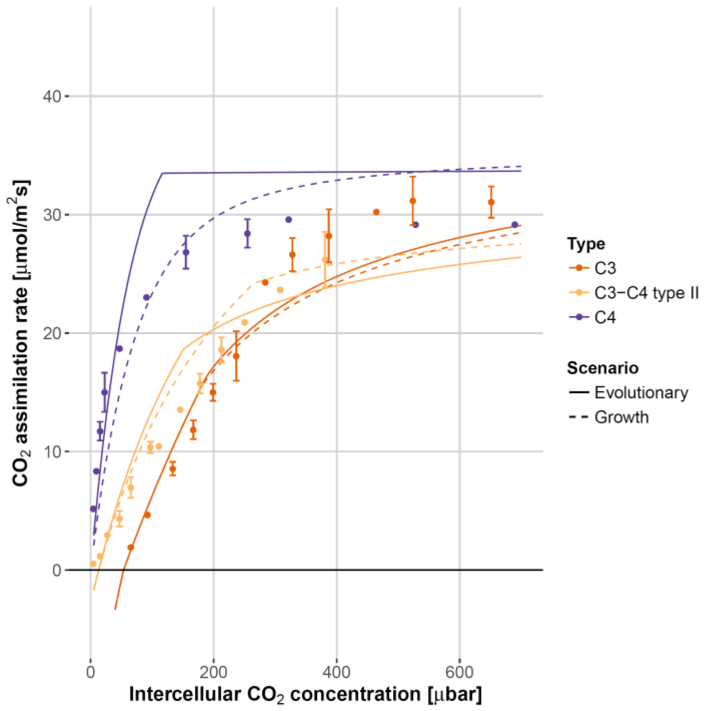

Fig. S3: Modeled results based on the evolutionary scenario (solid lines) and the model assuming optimal adaptation to the experimental growth conditions (dashed lines) describe the measured data (dots ± SE) for *F. bidentis* (C_4). Related to Fig. 2a, but the A-C_i curve is measured for plants grown at the low CO_2 level of 180 µbar (data from Vogan and Sage[1]). Missing error bars result from unknown empirical errors. See Table S9 for the residual sum of squares. Figure created using R 4.0 (https://www.R-project.org/).

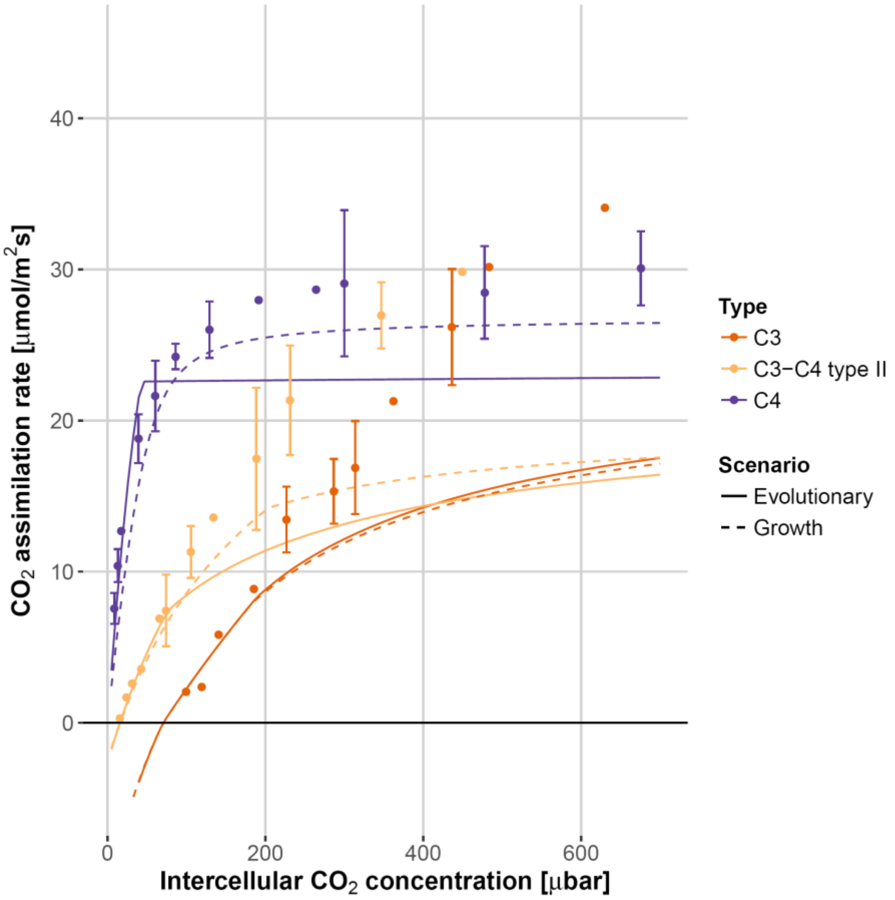

Fig. S4: Modeled results based on the evolutionary scenario (solid lines) and the model assuming optimal adaptation to the experimental growth conditions (dashed lines) describe the measured data (dots ± SE) for *F. bidentis* (C_4). Related to Fig. 2a, but the A-C_i curve is measured at 40°C and for plants grown at the low CO_2 level of 180 µbar (data from Vogan and Sage[1]). Missing error bars result from unknown empirical errors. See Table S9 for the residual sum of squares. Figure created using R 4.0 (https://www.R-project.org/).

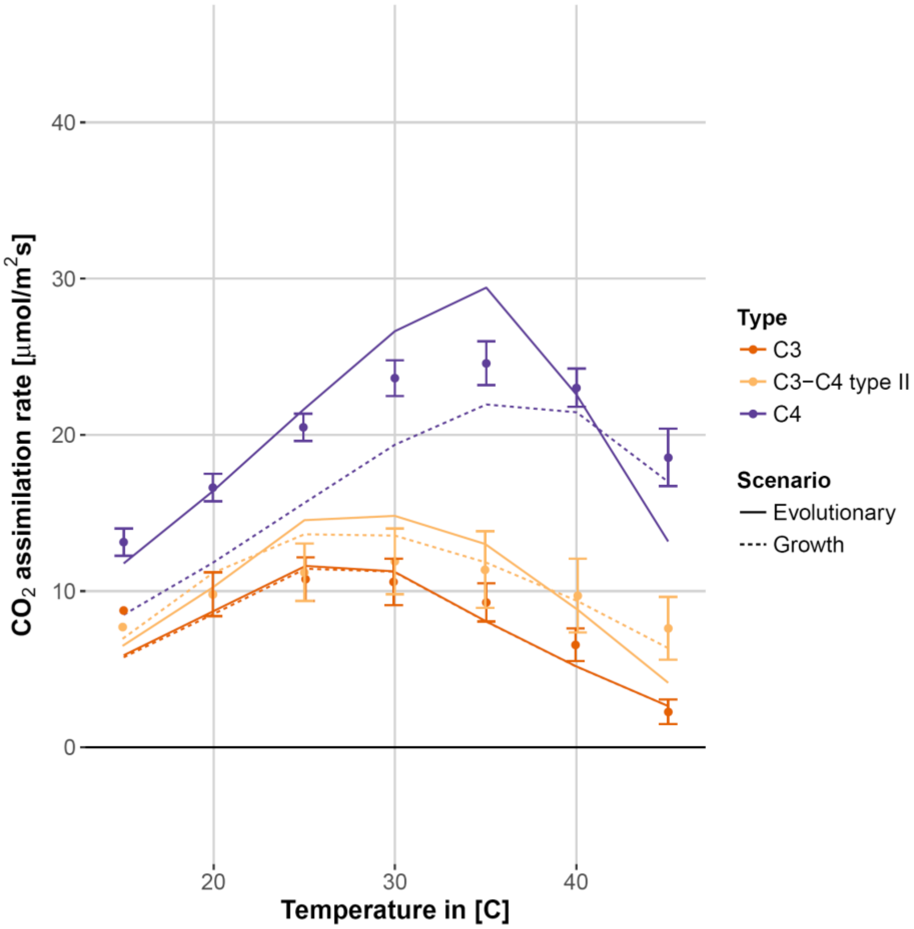

Fig. S5: Modeled results for temperature responses in the evolutionary scenario (solid lines) describe the measured data (dots ± SE) better than the model assuming optimal adaptation to the experimental growth conditions (dashed lines) for *F. bidentis* (C_4). Related to Fig. 2b, the A-Temperature curve is measured for plants grown at the low CO_2 level of 180 µbar (data from Vogan and Sage[1]). Missing error bars result from unknown empirical errors. See Table S9 for the residual sum of squares. Figure created using R 4.0 (https://www.R-project.org/).

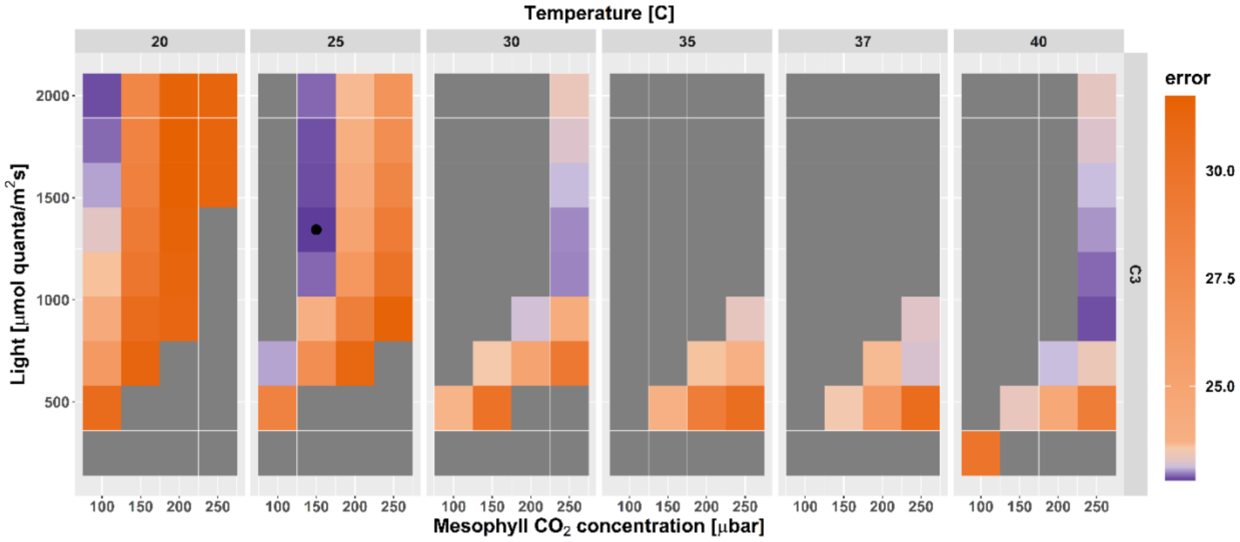

Fig. S6: Discrepancy between measured and modeled A-C_i curves of *F. robusta* (C_3) across diverse environments assuming no phosphate-limitation. Related to Figure 5. The deviation between model predictions and measurements (‘error’) is defined as the mean squared residuals of all measured curves (data from Vogan and Sage[1]). The black dot indicates the environment that best explains the experimental data. To make this analyse comparable with the C_4 analysis, the nitrogen allocation and the CO_2 assimilation rate are included in the error calculation. Here, the nitrogen allocation is considered by including an empirically determined ratio of maximal electron transport rate per Rubisco activity of 2±0.6 [4]. The grey areas indicate that the modeled nitrogen allocation cannot satisfy this ratio and that these points should not be used for inference. Figure created using R 4.0 (https://www.R-project.org/).

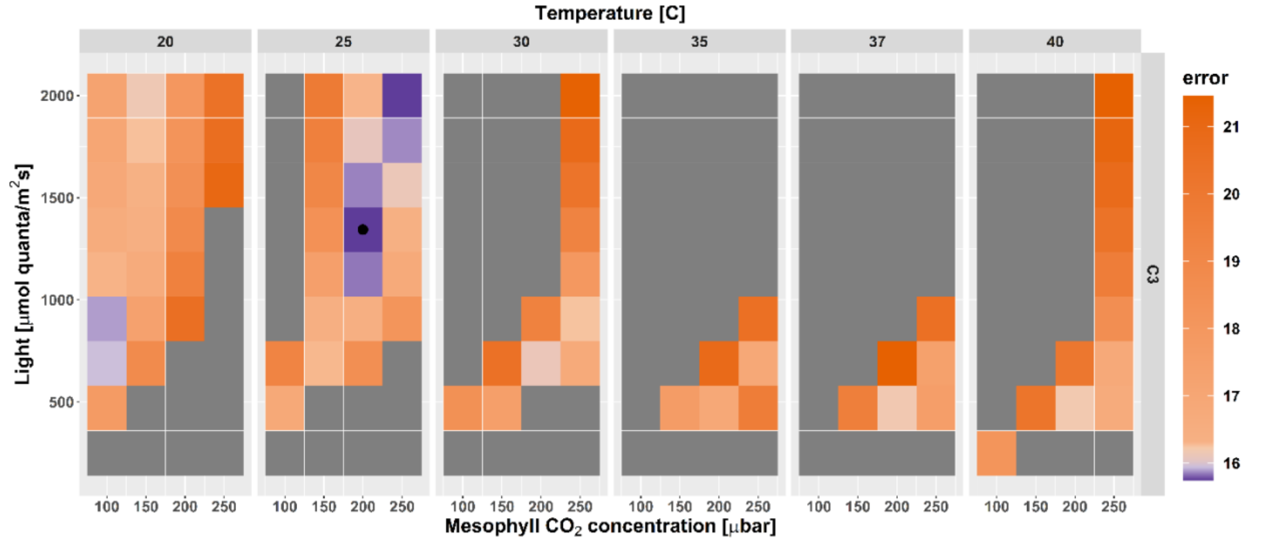

Fig. S7: Discrepancy between measured and modeled A-C_i curves of *F. robusta* (C_3) across diverse environments assuming phosphate-limitation for intercellular CO_2 levels above 400 µbar at 30°C and 500 µbar at 40°C for plants grown at 380 µbar atmospheric CO_2. Related to Figure 5. The deviation between model predictions and measurements ('error') is defined as the mean squared residuals of all measured curves from Ref. 1. The black dot indicates the environment that best explains the experimental data. To make this analyse comparable with the C_4 analysis, the nitrogen allocation and the CO_2 assimilation rate are included in the error calculation. Here, the nitrogen allocation is considered by including an empirically determined ratio of maximal electron transport rate per Rubisco activity of 2±0.6.[4] The grey areas indicate that the modeled nitrogen allocation cannot satisfy this ratio and that these points should not be used for inference. Figure created using R 4.0 (https://www.R-project.org/).

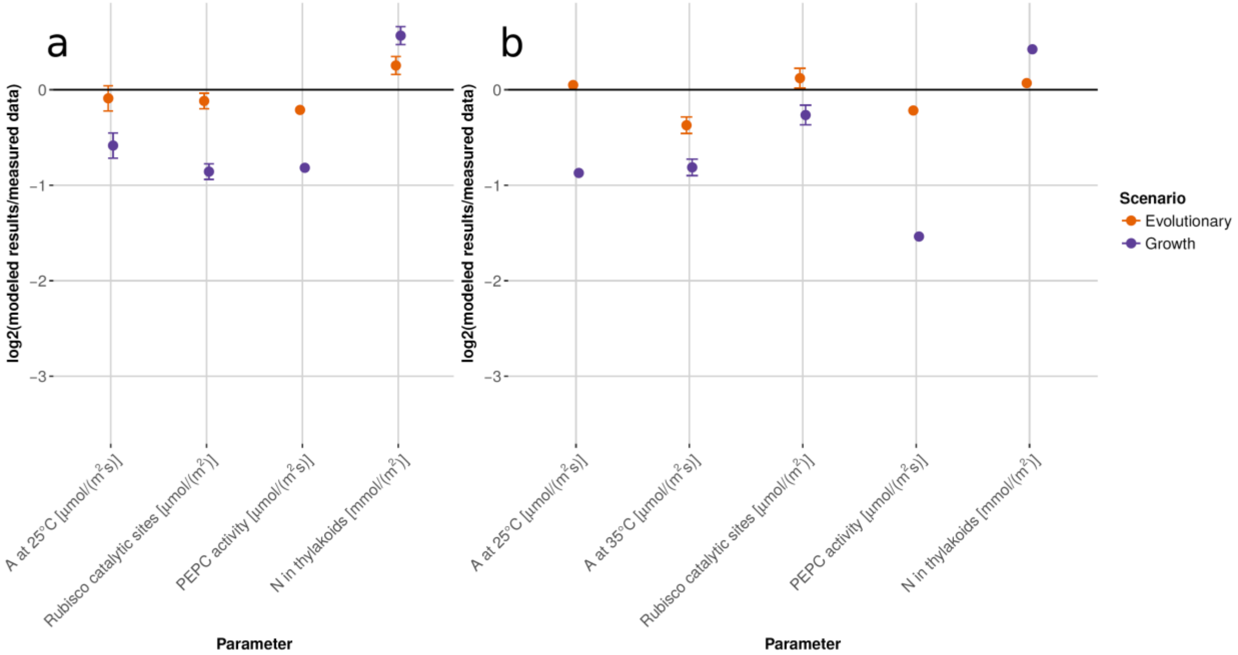

Fig. S8: A detailed analysis of resource allocation and physiology in *F. bidentis* (C_4) shows a good agreement between experimental data [3] and model results based on the evolutionary scenario (orange dots). Related to Figure 4. Alternative model results assuming optimal phenotypic adaptation to the growth scenario consistently show higher disagreement with the data (purple dots). Values are mean $\log_2$(modeled results/measured data) ± SE. (a) Plants grown at 25°C (b) Plants grown at 35°C. The *in vivo* PEPC activity is considered. A = net CO_2 assimilation rate; N = nitrogen. Figure created using R 4.0 (https://www.R-project.org/).

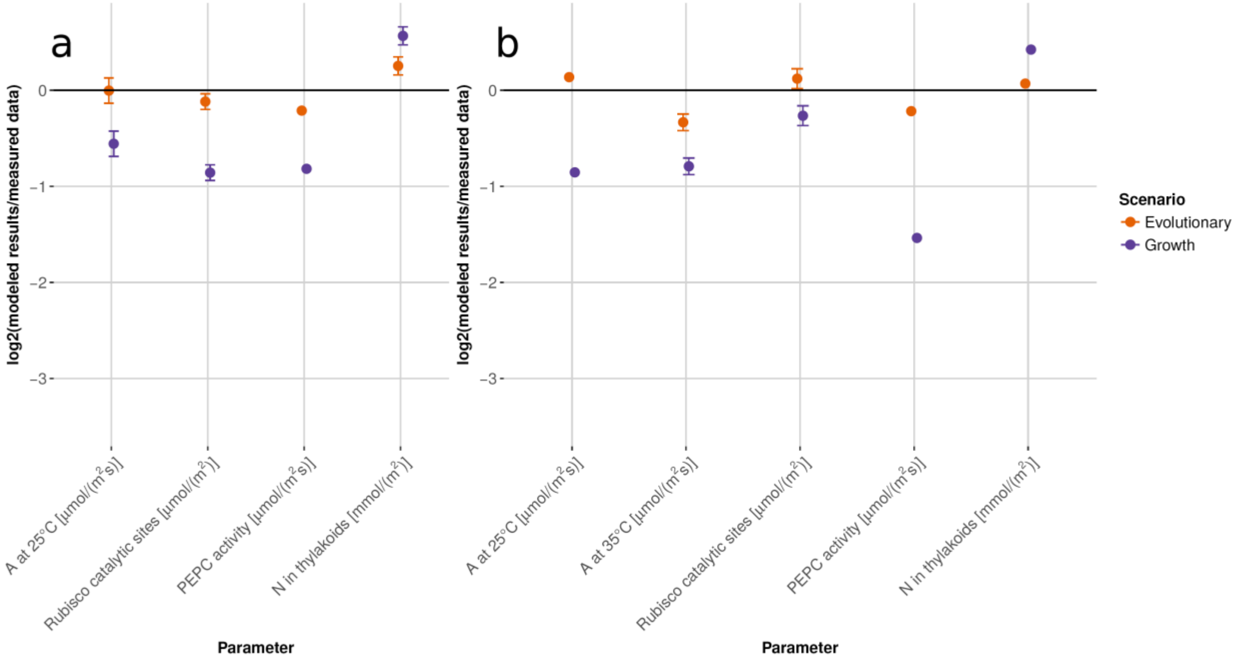

Fig. S9: A detailed analysis of resource allocation and physiology in *F. bidentis* (C_4) shows a good agreement between experimental data[3] and model results based on the evolutionary scenario (orange dots). Related to Figure 4. Alternative model results assuming optimal phenotypic adaptation to the growth scenario consistently show higher disagreement with the data (purple dots). Values are mean log_2(modeled results/measured data) ± SE. (a) Plants grown at 25°C (b) Plants grown at 35°C. It is assumed that energy allocation, including the proportion of linear electron transport, can adapt to the experimental measurement conditions. A = net CO_2 assimilation rate; N = nitrogen. Figure created using R 4.0 (https://www.R-project.org/).

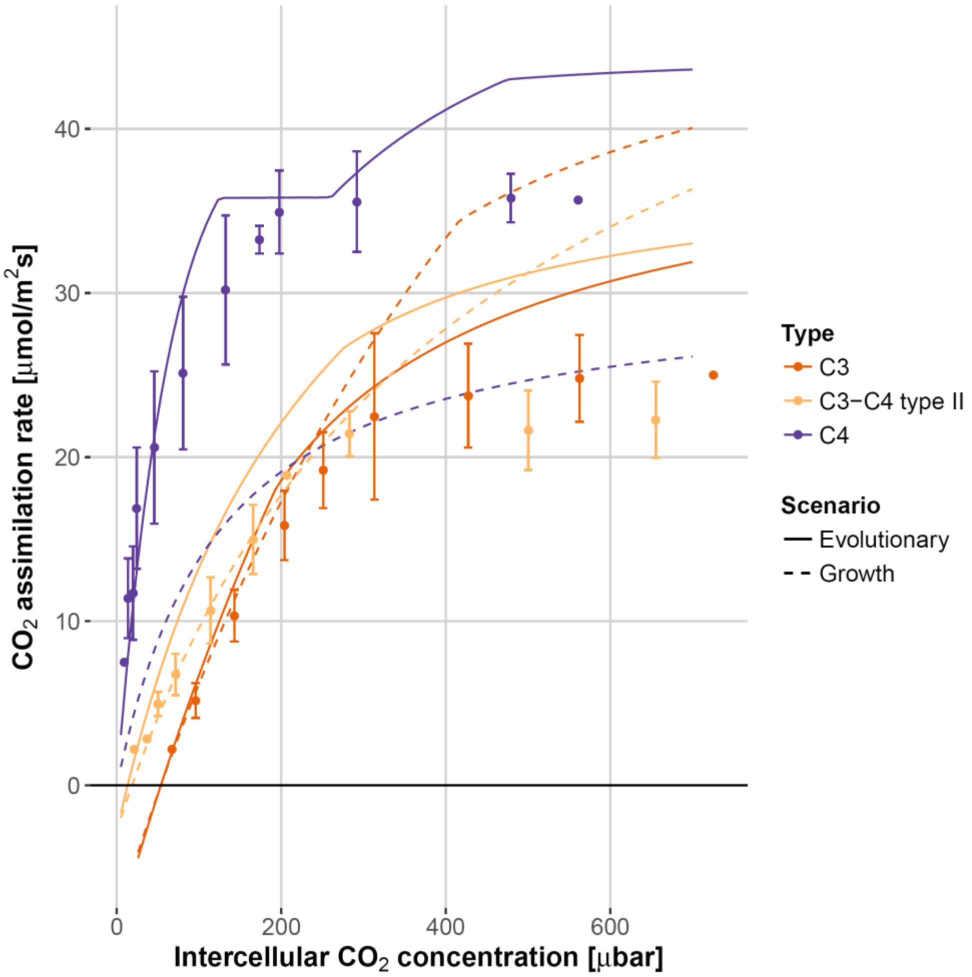

Fig. S10: Modeled results based on the evolutionary scenario (solid lines) and the model assuming optimal adaptation to the experimental growth conditions (dashed lines) describe the measured data (dots ± SE) for *F. bidentis* (C_4) grown at 380 µbar atmospheric CO_2 and measured at 30°C (data from Vogan and Sage[1]). Related to Figure 2. It is assumed that energy allocation, including the proportion of linear electron transport, can adapt to the experimental measurement conditions. Figure created using R 4.0 (https://www.R-project.org/).

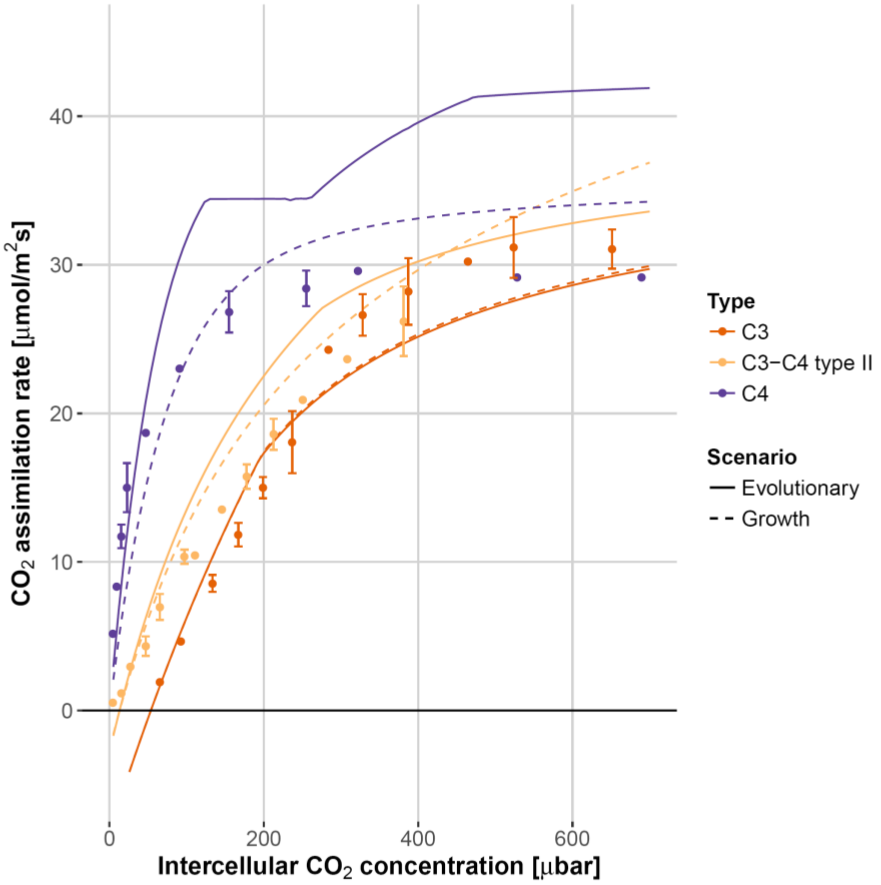

Fig. S11: Modeled results based on the evolutionary scenario (solid lines) and the model assuming optimal adaptation to the experimental growth conditions (dashed lines) describe the measured data (dots ± SE) for *F. bidentis* (C_4) grown at 180 µbar atmospheric CO_2 and measured at 30°C (data from Vogan and Sage[1]). Related to Figure 2. It is assumed that energy allocation, including the proportion of linear electron transport, can adapt to the experimental measurement conditions. Figure created using R 4.0 (https://www.R-project.org/).

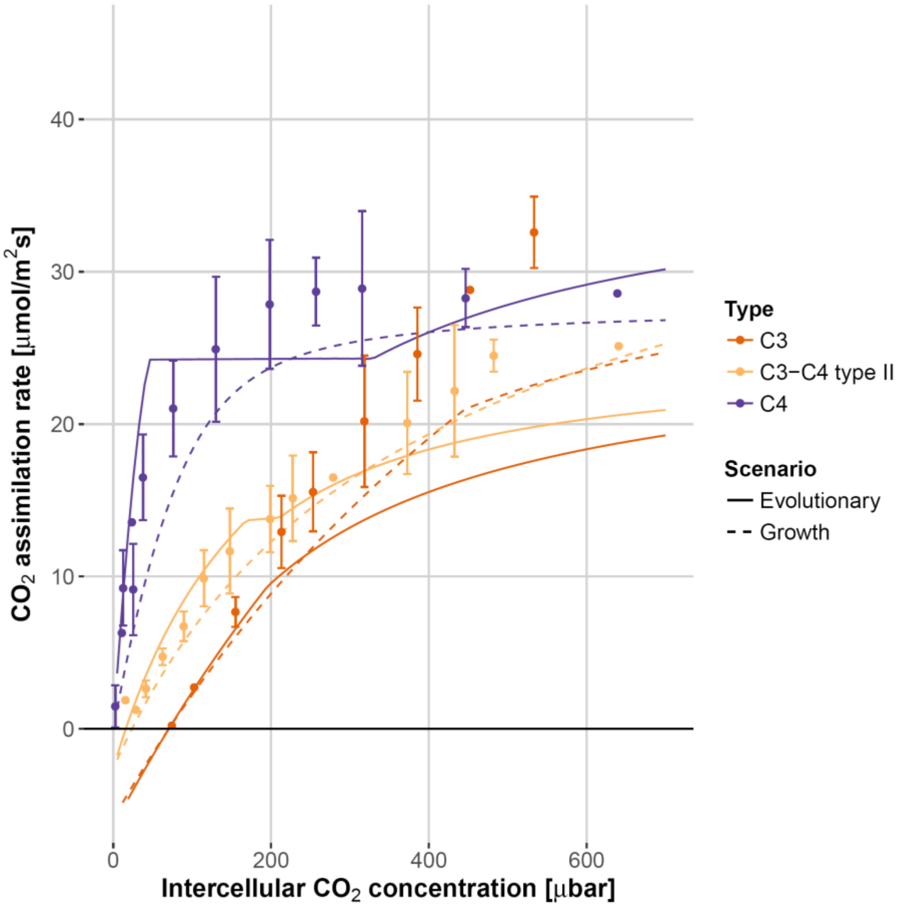

Fig. S12: Modeled results based on the evolutionary scenario (solid lines) and the model assuming optimal adaptation to the experimental growth conditions (dashed lines) describe the measured data (dots ± SE) for *F. bidentis* (C_4) grown at 380 µbar atmospheric CO_2 and measured at 40°C (data from Vogan and Sage[1]). Related to Figure 2. It is assumed that energy allocation, including the proportion of linear electron transport, can adapt to the experimental measurement conditions. Figure created using R 4.0 (https://www.R-project.org/).

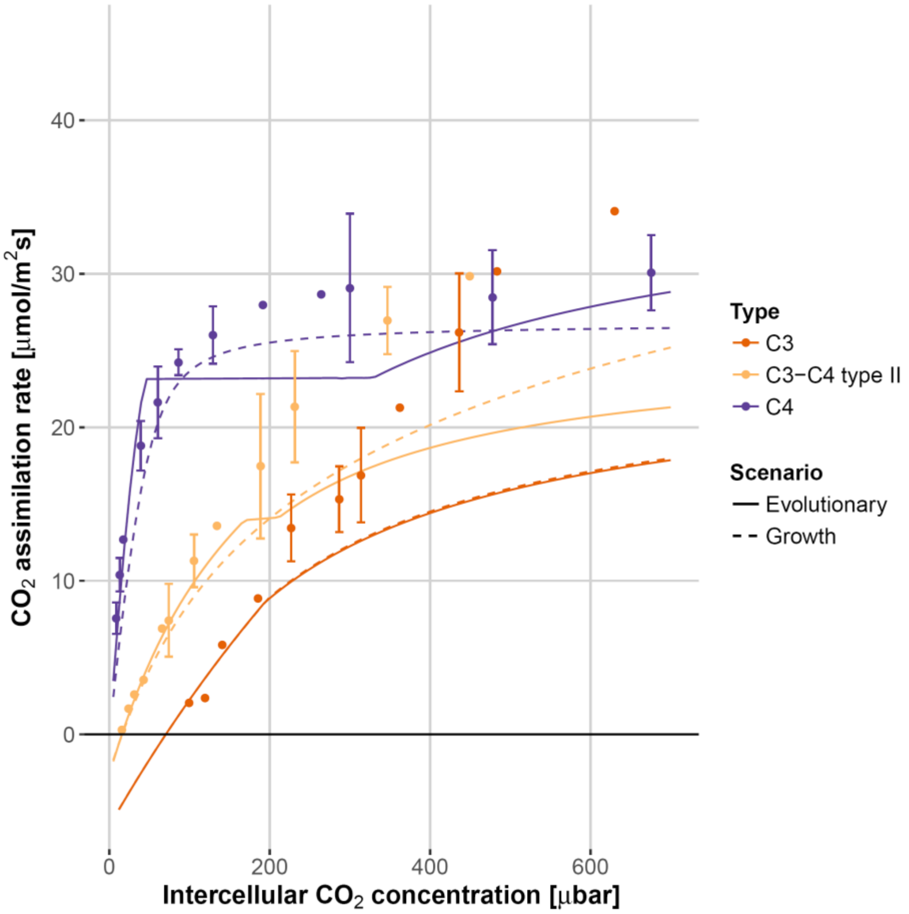

Fig. S13: Modeled results based on the evolutionary scenario (solid lines) and the model assuming optimal adaptation to the experimental growth conditions (dashed lines) describe the measured data (dots ± SE) for *F. bidentis* (C_4) grown at 180 µbar atmospheric CO_2 and measured at 40°C (data from Vogan and Sage[1]). Related to Figure 2. It is assumed that energy allocation, including the proportion of linear electron transport, can adapt to the experimental measurement conditions. Figure created using R 4.0 (https://www.R-project.org/).

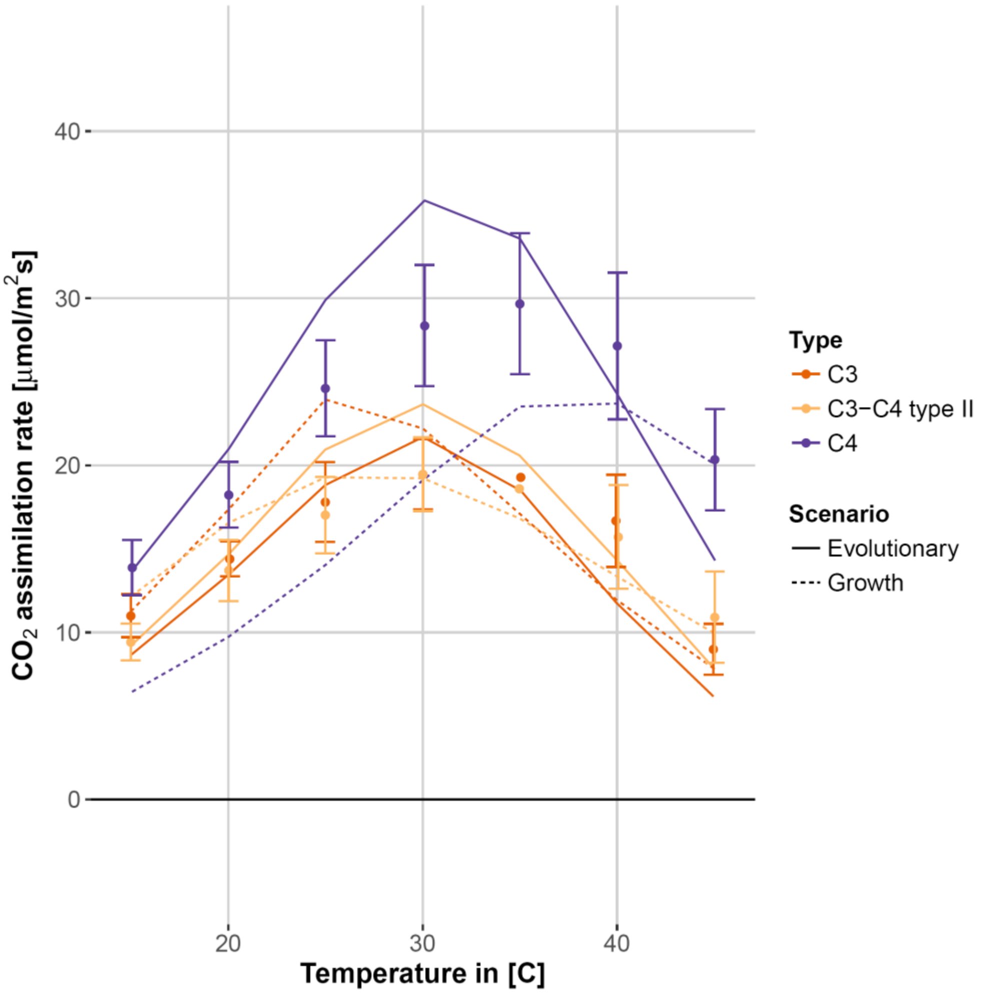

Fig. S14: Modeled results based on the evolutionary scenario (solid lines) and the model assuming optimal adaptation to the experimental growth conditions (dashed lines) describe the measured data (dots ± SE) for *F. bidentis* (C_4) grown at 380 µbar atmospheric CO_2 (data from Vogan and Sage[1]). Related to Figure 2. It is assumed that energy allocation, including the proportion of linear electron transport, can adapt to the experimental measurement conditions. Figure created using R 4.0 (https://www.R-project.org/).

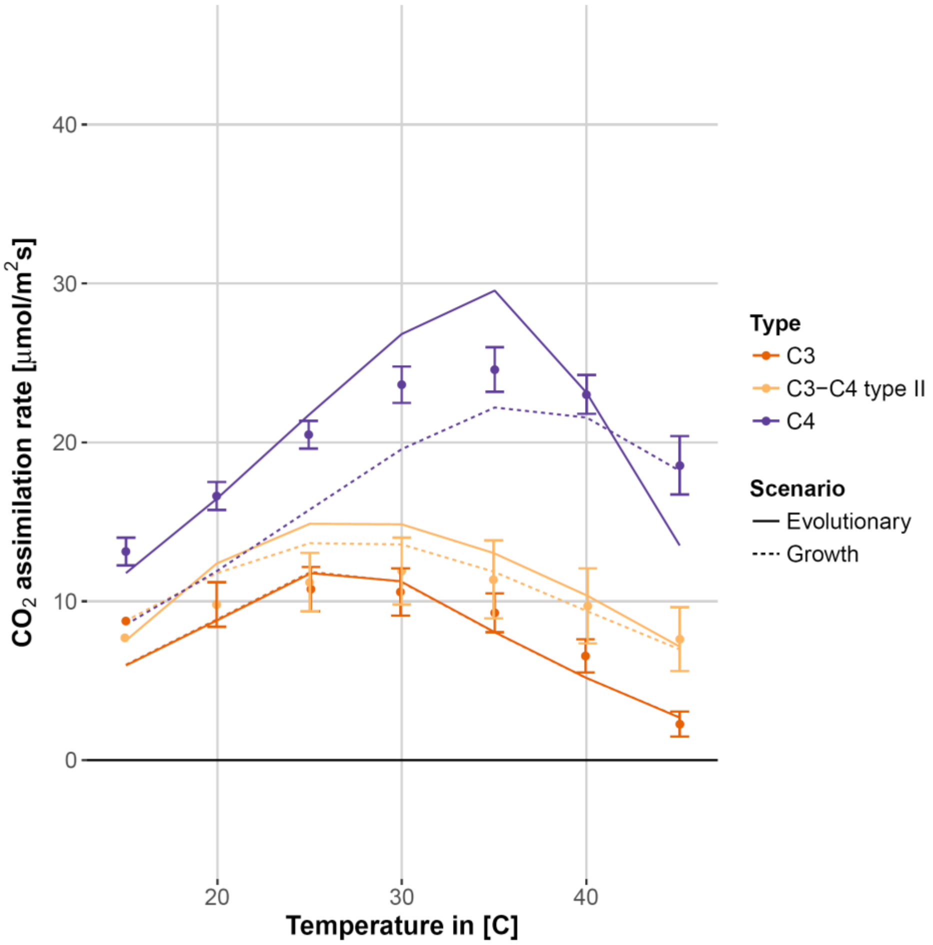

Fig. S15: Modeled results based on the evolutionary scenario (solid lines) and the model assuming optimal adaptation to the experimental growth conditions (dashed lines) describe the measured data (dots ± SE) for *F. bidentis* (C_4) grown at 180 µbar atmospheric CO_2 (data from Vogan and Sage[1]). Related to Figure 2. It is assumed that energy allocation, including the proportion of linear electron transport, can adapt to the experimental measurement conditions. Figure created using R 4.0 (https://www.R-project.org/).

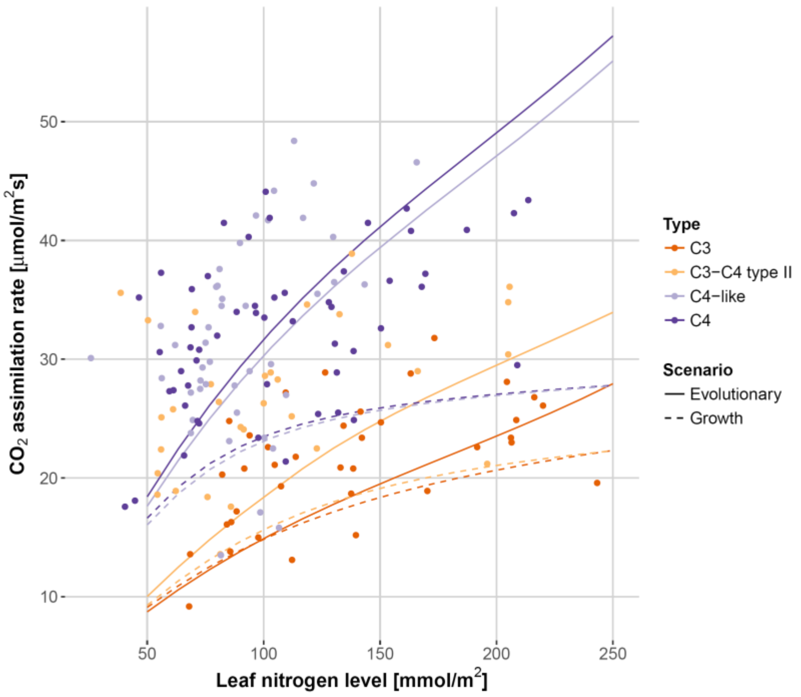

Fig. S16: The dependence of the CO_2 assimilation rate on leaf nitrogen levels for various *Flaveria* species is consistent with model results based on optimality in the evolutionary scenario (solid lines). For C_3-C_4 intermediate, C_4-like, and C_4 plants, these results outperform the ones assuming optimal phenotypic adaptation to the experimental growth conditions (dashed lines). The modeled species are *F. pringlei* (C_3), *F. floridana* (C_3-C_4), *F. palmeri* (C_4-like), and *F. bidentis* (C_4) (data from Vogan and Sage[2]). Related to Figure 3. It is assumed that energy allocation, including the proportion of linear electron transport, can adapt to the experimental measurement conditions. Figure created using R 4.0 (https://www.R-project.org/).

Supplementary Tables

Table S1: Model parameterization for different species of *Flaveria*. Related to Table 2. β, fraction of Rubisco expressed in the mesophyll cell [fraction]; k_{ccat}, the maximal turnover rate of Rubisco [s^{-1}]; V_{pmax}, the empirical maximal C_4 cycle activity determined by the PEPC activity [µmol m^{-2} s^{-1}]; K_p, Michaelis constant of PEPC [µbar]; g_s, bundle sheath conductance [µmol m^{-2} s^{-1}]; ξ, fraction of mesophyll cell derived photorespiration in the bundle sheath cells. All parameters are taken from Heckmann et al.[5], except for the maximal Rubisco turnover rate which is taken from Kubien et al.[6]. The maximal PEPC activity of the C_4 species is assumed to be unlimited, i.e., the upper bound is set to 1000.

Species	Photosynthetic types	β	k_{ccat}	V_{pmax}	K_p	g_s	ξ
F. pringlei	C_3	0.95	3.11	0	200	0.015	0
F. robusta	C_3	0.95	3.11	0	200	0.015	0
F. floridana	C_3-C_4 type II	0.52	3.19	40.2	200	0.015	0.79
F. ramosissima	C_3-C_4 type II	0.65	2.77	40.2	200	0.015	0.58
F. palmeri	C_4-like	0.068	3.54	75.9	80	0.001	0.97
F. bidentis	C_4	0.008	4.16	1000	80	0.001	0.96

Table S2: Lower and upper bounds for the model parameters subject to numerical optimization. Related to Table 2.

Parameter	Lower bound	Upper bound
Proportion of LET (*p*)	$1.0x10^{-10}$	$1 – 1.0x10^{-10}$
Fraction of nitrogen invested into the C_4 cycle (n_{C4})	0	The measured maximal PEPC activity is used to calculate the maximal investment into the C_4 cycle (n_{C4}^{max}, Eqn S3)
Fraction of nitrogen invested into the thylakoids (n_{Jmax})	The nitrogen investment into the CET is independent of *p*, therefore the bound for n_{Jmax} (n_{Jmax}^{min}) can be set as fixed to the nitrogen requirements of the CET (Eqn S4).	1
Fraction of nitrogen invested into Rubisco (n_{Etot})	0	1

Table S3: The parameters of the temperature-dependent model. Related to Table 2. The variable names for the temperature-dependent parameters are the same as in the main text and in the Supplementary text above. *E*, the activation energy [J mol^{-1}]; *H*, the deactivation energy [J mol^{-1}]; *S*, entropy factor [J mol^{-1}]; *R*, the universal gas constant [J mol^{-1}]; inac, indicates whether inactivation occurs.

Temperature-dependent variable	Parameter	Value	Source
V_{cmax}	E	7.543351e+04	fit to Ku *et al.* [7]
V_{cmax}	Inac	TRUE	
V_{cmax}	H	1.213043e+05	fit to Ku *et al.* [7]
K_c	E	4.175349e+04	fit to Ku *et al.* [7]
K_c	Inac	FALSE	
K_o	E	5.314215e+04	fit to Ku *et al.* [7]
K_o	Inac	FALSE	
γ_*	E	2.166348e+04	fit to Ku *et al.* [7]
γ_*	Inac	FALSE	
V_{pmax}	E	7.0373e4	Massad *et al.* [8]
V_{pmax}	Inac	FALSE	
K_p	E	5.455e4	Chen *et al.* [9]
K_p	Inac	FALSE	
g_s	E	1.898e4	fit to Tamimi *et al.* [10]
g_s	Inac	FALSE	
J_{max}	E	77900	Massad *et al.* [8]
J_{max}	Inac	TRUE	
J_{max}	S	62	Massad *et al.* [8]
J_{max}	H	191929	Massad *et al.* [8]

Table S4: The parametrization of the evolutionary environment. Related to Figures 2-4. All conditions consider current atmospheric O_2 concentrations, which equals 200 mbar mesophyll O_2 concentration. See Table S8 for the corresponding mesophyll CO_2 concentration.

Environment	Atmospheric CO_2 concentration [µbar]	Temperature [C]	Light intensity [µmol m^{-2} s^{-1}]	Leaf nitrogen level [mmol m^{-2}]
Evolutionary environment	280	30	1750	According the empirically determined value of the corresponding experiment

Table S5: The parametrization of the experimental measurement and experimental growth environments of the Vogan and Sage Sage[1] data set. Related to Figures 2-4. “All” indicates that all photosynthetic types and the corresponding leaf nitrogen levels were considered. “Various” indicates that a range of values are tested. All conditions consider current atmospheric O_2 concentrations, which equals 200 mbar mesophyll O_2 concentration. See Table S8 for the corresponding mesophyll CO_2 concentration.

	Environment	Photosyn-thetic type	Atmospheric CO_2 concentra-tion [µbar]	Temperature [C]	Light intensity [µmol m^{-2} s^{-1}]	Leaf nitrogen level [mmol m^{-2}]
Growth conditions	“Current” growth conditions of Vogan and Sage [1]	C_3	380	37	561	187
		C_3-C_4	380	37	561	150
		C_4	380	37	561	136
	“Low” CO_2 conditions of Vogan and Sage [1]	C_3	180	37	561	167
		C_3-C_4	180	37	561	154
		C_4	180	37	561	127
Measurement conditions	A-C_i curves	all	various	30 and 40	1500	According the empirically determined value
	A-Temp. curves	all	180 or 380	various	1500	According the empirically determined value

Table S6: The parametrization of the experimental measurement and experimental growth environments of the Vogan and Sage[2] data set. Related to Figure 3. "Various" indicates that a range of values are tested. All conditions consider current atmospheric O_2 concentrations, resulting in 200 mbar mesophyll O_2 concentration. See Table S8 for the corresponding mesophyll CO_2 concentration.

Environment	Atmospheric CO_2 concentration [µbar]	Temperature [C]	Light intensity [µmol m^{-2} s^{-1}]	Leaf nitrogen level [mmol m^{-2}]
Growth conditions	380	30	554	various
Measurement conditions	380	30	2000	According the empirically determined value

Table S7: The parametrization of the experimental measurement and experimental growth environments of Dwyer et al.[3]. Related to Figure 4. See Table S8 for the corresponding mesophyll CO_2 concentration.

	Environment	Atmospheric CO_2 concentration [µbar]	Temperature [C]	Light intensity [µmol m^{-2} s^{-1}]	Leaf nitrogen level [mmol m^{-2}]
Growth conditions	Moderate temperature condition of Dwyer et al. [3]	380	25	550	183
	High temperature condition of Dwyer et al. [3]	380	35	550	153
Measurement condition		665	25 or 35	2000	According the empirically determined value

Table S8: Mesophyll CO_2 concentration as a function of atmospheric CO_2 concentration and photosynthetic type. Related to Figures 2-4. It is assumed that C_4-like and C_4 species show the same mesophyll CO_2 concentration. Dashes indicate that this value is not required.

	Atmospheric CO_2 concentration [µbar]			
	180	280	380	665
C_3	120	170	215	-
C_3-C_4	95	140	190	-
C_4-like	60	115	170	-
C_4	60	115	170	300

Table S9: In C_4 and C_4-like plants, the evolutionary scenario shows significantly smaller squared residuals compared to the growth scenario. Related to Figures 2-3 and S2-S5. The residual sum of squares for the evolutionary and growth scenario, each photosynthetic type, and all measured curves of Vogan and Sage[1,2] are presented.

		C_3	C_3-C_4 intermediate	C_4-like	C_4
Evolutionary scenario	Fig. 2a	96	68		80
	Fig. 2b	46	48		135
	Fig. 3	599	2003	837	1711
	Supplementary Fig. S2	550	326		203
	Supplementary Fig. S3	92	88		222
	Supplementary Fig. S4	744	551		218
	Supplementary Fig. S5	14	37		65
Growth scenario	Fig. 2a	477	236		1783
	Fig. 2b	72	20		387
	Fig. 3	585	2783	1990	3241
	Supplementary Fig. S2	269	53		276
	Supplementary Fig. S3	113	57		182
	Supplementary Fig. S4	784	375		107
	Supplementary Fig. S5	15	13		98

Table S10: The modeled and measured data of chlorophyll [µmol m-2], PSII [µmol m-2], and cytochrome f [µmol m^{-2}] of *F. bidentis* (C_4). Related to Figure 4. The plants were grown at 25°C or 35°C (data from Dwyer et al.[3]).

	Growth at 25°C		Growth at 35°C	
	modeled	measured	modeled	measured
Photosystem II [µmol m^{-2}]	1.51	1.24	1.46	1.28
Chlorophyll [µmol m^{-2}]	602	499	585	533
Cytochrome f [µmol m^{-2}]	1.56	0.87	1.35	0.80

Table S11: Distribution parameters used to generate the random parameter sets for the sensitivity analysis shown in Fig. S1. Related to Figures 2-4. For each considered variable, mean and standard deviation (SD) of the sampled normal distribution are shown.

	mean	SD
The mesophilic CO_2 concentration (C_m, which is calculated by scaling the considered standard C_m)	1	0.25
The proportion of nitrogen invested into the PCO enzymes that can be saved by preventing high photorespiratory rates	1	0.167
The relationship between cytochrome f and maximal electron transport of the LET (cyt_{Jmax})	172	24
The scaling factor for the maximal electron transport rate of the CET relative to that of the LET ($Jmax_{CL}$)	3	0.67
The empirical curvature factor (Θ)	0.7	0.083

Supplementary Methods

Method S1: Photosynthetic leaf nitrogen level

To calculate the CO_2 assimilation rate, we focus on the photosynthetic nitrogen pool (N_{ps}, [µmol m^{-2}]). N_{ps} is calculated as a fraction of total leaf nitrogen (N_t, [µmol m^{-2}]) based on phenomenological observations according to Eqn S1, which comprises measured values for the investment into Rubisco ($n_{Rubisco}$, [fraction]) of 12%, and the investment into the thylakoids (n_{fit}, [fraction]) of C_3 plants [1,2]. n_{fit} represents a fit of the proportion of nitrogen invested into the thylakoids as a function of N_t, based on the data of Vogan and Sage [2].

$$N_{ps} = \left(n_{Rubisco} + n_{fit}\right) \cdot N_t \tag{S1}$$

with

$$n_{fit} = \left(\frac{50.38 - 0.270 \cdot N_t \cdot 10^{-3} + 0.0005035 \cdot (N_t \cdot 10^{-3})^2}{100}\right)$$

Note that, while we use $n_{Rubisco}$ and n_{fit} to estimate N_{ps}, nitrogen investment into light reactions and Calvin-Benson cycle are free variables in the optimization procedure described in the Methods section of the main text. We further assume a nitrogen investment into the photorespiratory enzymes of 13.8%, as suggested by Zhu et al. [11] for a 'typical' C_3 plant. To account for the reduced enzyme requirements of the photorespiratory cycle, we assume that N_{ps} increases by 10% in plants that show sufficient C_4 cycle activity; in our analyses, this applies to the C_3-C_4 intermediate, C_4-like, and C_4 species.

Method S2: Chlorophyll content

The leaf chlorophyll content is used to incorporate chlorophyll-based measurements of nitrogen demands of PSI, PSII, the LHC, and cytochrome f. Chlorophyll content (Chl, [µmol m^{-2}]) is calculated based on an empirical factor [1] that relates the amount of nitrogen invested into thylakoids (n_{fit} N_t, Eqn S1) to the amount of chlorophyll in C_3 plants (n_{Chl}, [factor]). n_{Chl} is 0.0158887:

$$Chl = n_{fit} \cdot N_t \cdot n_{Chl} \tag{S2}$$

The response of chlorophyll content to leaf nitrogen does not differ significantly between different photosynthetic types in *Flaveria* [2].

Method S3: Details about the optimization procedure of resource allocation

To restrict the results of the optimization of CO_2 assimilation rates to biologically relevant resource allocation patterns, we enforce a set of constraints. The relative contributions of the components of the following three pools have to sum up to one in each pool: (1) the nitrogen investments into the enzymes of the Calvin-Benson cycle in the mesophyll or bundle sheath cells, the C_4 cycle, or the thylakoids including the cost of the linear and cyclic electron transport; (2) the ATP investments into the C_4 cycle, Rubisco activity in mesophyll and bundle sheath cells, as well as the non-photochemical quenching (which is nearly zero, in case of optimality); and (3) the NADPH investments into the NADPH-relevant sub-pools. Further constraints ensure that the electron transport rate does not exceed the rate sustained by current irradiance and that the production of ATP and NADPH has to fulfill or exceed the respective consumption. When calculating the light- and enzyme-limited CO_2 fixation rate in the bundle sheath cells, the resulting quadratic equations can only be solved if the radicands are larger or equal to zero (Eqns S10 and S12). Table S2 shows the lower and upper bounds of the parameters that are optimized. All parameters represent fractions, therefore all lower and upper bounds have to be between zero and one. The lower bound of p is set close to zero to avoid division by zero. The upper bound of n_{C4} (n_{C4}^{max}) is based on the empirical maximal C_4 cycle activity (V_{pmax}^{emp}) and the photosynthetic nitrogen level (Eqn S3).

$$n_{C4}^{max} = \frac{V_{pmax}^{emp}\left(\frac{MW^*{}_{PPDK}}{kcat_{PPDK}} + \frac{MW^*{}_{PEPC}}{kcat_{PEPC}}\right)}{N_{ps}} \quad \text{(S3)}$$

The lower bound of n_{Jmax} (n_{Jmax}^{min}) ensures that the nitrogen requirements of PSI, PSII, and LHC are met. As n_{Jmax} depends on the photosynthetic nitrogen level and the proportion of the LET, a parameter to be optimized, this dependency results in a bound (Eqn S4, also see Eqn 13) and a constraint that is relevant during the optimization procedure (Eqn S5, also see Eqn 9 and 13). n_{Jmax}^{min} is calculated for each scenario and photosynthetic type separately.

$$n_{Jmax}^{min} = \left(\frac{Chl}{N_{ps}}\right)\left(PSI \cdot pI_N \cdot pI_{Chl} \cdot 10^{-3} + \left(\frac{1000 - PSI \cdot pI_{Chl}}{l_{Chl}}\right) \cdot l_N \cdot l_{Chl} \cdot 10^{-3}\right) \quad \text{(S4)}$$

$$n_{Jmax} \geq c_{Jmax}^{min} \quad \text{(S5)}$$

with

$$c_{Jmax}^{min} = max\,(c_{Jmax}^{LET}, c_{Jmax}^{CET})$$

$$c_{Jmax}^{LET} = (PSII \cdot pII_N \cdot pII_{Chl} \cdot 10^{-3} + PSI_{LET} \cdot pI_N \cdot pI_{Chl} \cdot 10^{-3} + LHC_{LET} \cdot l_N \cdot l_{Chl} \cdot 10^{-3})\left(\frac{Chl}{N_{ps} \cdot p}\right)$$

$$c_{Jmax}^{CET} = \left(\frac{Chl}{N_{ps}(1-p)}\right)(PSI_{CET} \cdot pI_N \cdot pI_{Chl} \cdot 10^{-3} + LHC_{CET} \cdot l_N \cdot l_{Chl} \cdot 10^{-3})$$

and with pII_N, pI_N, l_N, pII_{Chl}, pI_{Chl}, and l_{Chl} from the main text.

The optimization was implemented in the R environment [12], using the auglag-function of the package 'nloptr' [13]. The optimization algorithm can use various local solvers; we chose the derivative-free solver, 'COBYLA'. We adapted the parameters of the auglag-function as follows: (1) xtol_rel=1x10-100, i.e., we stop the optimization when all parameters changed by a proportion <1x10-100 in the last iteration; (2) localtol, the tolerance applied in the selected local solver, is set to 1x10-100; and (3) maxeval, the maximal number of optimization iterations, is set to 5x103. To ensure robust retrieval of the global optimum, up to 735 initial values are used for the optimization procedure. We use equidistant points that span the range of minimum and maximum bounds for each nitrogen pool (Table S2). As optimal energy allocation is a function of the nitrogen pools, this can lead to unrealistic values of energy demand in some initial points. We thus excluded points for which absolute sum of energy allocation fractions exceed 1000. For the proportion of the LET, there are seven initial values that cover the expected range of values (namely those are 0.5, 0.6, 0.65, 0.75, 0.85, 0.95, 0.99, see Yin and Struik [14]). Note that at the point of optimal resource allocation, the light- and enzyme-limited CO2 assimilation rates are equal, as otherwise resources could be shifted from the non-limiting to the limiting sector.

Method S4: ATP and NADPH requirements

The ATP and the NADPH requirements of the Calvin-Benson cycle (CBB), the photorespiratory pathway (PR), and the C_4 cycle are based on the work of von Caemmerer [15]. The variables are defined in the main text and in the Supplementary text above. Additionally, O represents the O_2 concentration in the considered cell type (O_m or O_s), and C represents C_m or C_s.

The ATP requirements of the Calvin-Benson cycle, the photorespiratory path, and the C_4 cycle are:

$$E_{ATP_{CBB,PR}}(O,C) = 3\, e_{ATP}\left(1 + \frac{7\,\gamma_* O}{3C}\right) \tag{S6}$$

$$E_{ATP_{C4}} = 2\, e_{ATP} \tag{S7}$$

NADPH is required for the CBB and the PR, but not for the C_4 cycle. The requirements are:

$$E_{NADPH_{CBB,PR}}(O,C) = 2\left(2 + \frac{4\,\gamma_* O}{C}\right) \tag{S8}$$

The number of electrons transported to generate one molecule of ATP is under discussion; for a discussion, see, e.g., Amthor [18]. We address these uncertainties by a factor that represents the ratio of electron transported per ATP in LET, which we set to e_{ATP} = 4/3 in this work. In *Flaveria*, this ratio is supported by Siebke *et al.* [19]. The energy requirements of the C_4 cycle are adequate for the C_4-subtypes that utilize NAD-malic enzyme or NADP-malic enzyme, whose ATP demand can be assumed to be equal. For the C_4-subtype that utilizes PEP carboxykinase, the energetic costs are different and currently unclear [15,20].

Method S5: Equations of the light-limited CO2 assimilation rate

The equations describing the ATP-limited case are derived from the C_3-C_4 model of von Caemmerer [15]. The variables are defined in the main text and in the Supplementary text above. Additional variables are (1) the CO_2 concentration in the mesophyll cell (C_m); (2) the O_2 concentration in the mesophyll cell (O_m); (3) the mitochondrial respiration in the mesophyll cell (R_m); and (4) the mitochondrial respiration in the bundle sheath cell (R_s). Note that the fraction of PSII activity in the bundle sheath cells is set to p.

Considering a variable electron to ATP ratio (e_{ATP}) results in the following equations for the rate of CO_2 fixation in the mesophyll and bundle sheath cell, respectively:

$$A_m = \frac{(C_m - \gamma_* O_m) J_{mc}}{3\, e_{ATP}\left(C_m + \frac{7\,\gamma_*\, O_m}{3}\right)} \tag{S9}$$

$$A_s = \frac{-b - \sqrt{b^2 - 4ac}}{2a} \tag{S10}$$

with

$$S = \frac{J_{mp}}{2\, e_{ATP}} + \frac{\xi \gamma_* O_m\, J_{mc}}{e_{ATP}(3\, C_m + 7\, \gamma_* O_m)}$$

$$a = \frac{1}{4}\left(3\, e_{ATP} - \frac{7\, e_{ATP}\, \gamma_*\, p}{0.047}\right)$$

$$b = \frac{1}{4}\left(3\, e_{ATP}\, R_s - 3\, e_{ATP}\, S - 3\, g_s\, C_m\, e_{ATP} - J_s - \frac{7\, R_s\, e_{ATP}\, p\, \gamma_*}{0.047} - 7\, g_s\, e_{ATP} \gamma_*\, O_m - \frac{J_s\, \gamma_*\, p}{0.047}\right)$$

$$c = \frac{1}{4}\left((S + g_s\, C_m)\, (J_s - 3\, R_s\, e_{ATP}) - g_s\, \gamma_*\, O_m (7\, R_s\, e_{ATP} + J_s\,)\right)$$

Since NADPH does not affect the C_4 cycle, its NADPH-limited rate, V_p, is at its maximal value, V_{pmax}. The equations of the NADPH-limited case are as follows:

$$A_m = \frac{\left(1 - \frac{\gamma_* O_m}{C_m}\right) J_{mc}}{4 + \frac{8\,\gamma_* O_m}{C_m}} \tag{S11}$$

$$A_s = \frac{-b + \sqrt{b^2 - 4ac}}{2a} \tag{S12}$$

with

$$S = g_s\, C_m + V_{pmax} + \left(\frac{\frac{\xi\, J_{mc}\, \gamma_*\, O_m}{C_m}}{4 + \frac{8\,\gamma_* O_m}{C_m}} \right)$$

$$a = -4 + \frac{8\, p\, \gamma_*}{0.047}$$

$$b = -4\, R_s + J_s + 4\, S + \frac{p\, \gamma_*}{0.047}(8\, R_s + J_s) + 8\, g_s\, O_m\, \gamma_*$$

$$c = S(4\, R_s - J_s) + g_s\, O_m\, \gamma_*(8\, R_s + J_s)$$

Method S6: Details about the temperature-dependent model

The values required to describe the temperature response of the Rubisco activity (V_{cmax}, [µmol m^{-2} s^{-1}]), the Rubisco specificity ($2\gamma_*$), and the Michaelis constants of Rubisco for CO_2 (K_c, [µbar]) and O_2 (K_o, [µbar]) were fitted to the data of Ku *et al.* [7] simultaneously with the number of Rubisco catalytic sites. We approximate the temperature response of g_s with the temperature response of the diffusion coefficient of CO_2 in water. Table S3 shows the parameters for each temperature-dependent variable.

Method S7: Sensitivity analysis

The following parameters are considered in the sensitivity analysis: (1) the mesophilic CO_2 concentration (C_m); (2) the proportion of nitrogen invested into photorespiration (PCO) enzymes that can be saved by preventing high photorespiratory rates; (3) the relationship between cytochrome f and maximal electron transport of the LET (cyt_{Jmax}, [mmol e$^-$(mmol cyt s)$^{-1}$]), which is only known for C_3 species; (4) the scaling factor for the maximal electron transport rate of the CET relative to that of the LET ($Jmax_{CL}$); and (5) the empirical curvature factor (Θ), for which different values are frequently used in the literature. 200 parameter sets were sampled from an uncorrelated multivariate normal distribution (see Table S11 for details). The mean of the normal distribution is set to the standard value (see Methods, Table S11), except for the proportion of nitrogen invested into the PCO enzymes that can be saved by preventing high photorespiratory

rates, this value is set to one. By that, we focus on the parameter sets that hypothesize the same photosynthetic nitrogen level for C_3 and C_4 plants. The standard deviation for each parameter is chosen such that on the one hand values that are discussed in the literature [21] and on the other hand uncertainties are potentially covered. We calculated the mean squared residuals (expressed as fractions of the experimental means) for the relevant Dwyer *et al.* [3] data for plants grown at 25°C compared to data predicted from optimal resource allocation to either the growth or to the evolutionary scenario (Fig. S1). For each of the 200 random parameter sets, the predictions based on the evolutionary scenario led to lower error than those based on the experimental growth environment (see Fig. S1).

Method S8: Statistical information about the data set of Vogan and Sage[2]

Due to computational limitations, a limited number of leaf nitrogen levels can be used to calculate the resource allocation for the data set of Vogan and Sage [2] (Fig. 3). We considered 16 leaf nitrogen levels for the calculation of the resource allocation and CO_2 assimilation rates. We inferred the CO_2 assimilation rates required for the remaining leaf nitrogen levels from linear interpolation between the two closest leaf nitrogen levels. For the statistical analysis, the data of the modeled species, *F. pringlei* (C_3), *F. floridana* (C_3-C_4), *F. palmeri* (C_4-like), and *F. bidentis* (C_4), were considered.

Supplementary References

1 Vogan, P. J. & Sage, R. F. Effects of low atmospheric CO_2 and elevated temperature during growth on the gas exchange responses of C_3, C_3-C_4 intermediate, and C_4 species from three evolutionary lineages of C_4 photosynthesis. *Oecologia* **169**, 341-352, doi:10.1007/s00442-011-2201-z (2012).

2 Vogan, P. J. & Sage, R. F. Water-use efficiency and nitrogen-use efficiency of C_3-C_4 intermediate species of *Flaveria* Juss. (Asteraceae). *Plant, Cell & Environment* **34**, 1415-1430, doi:10.1111/j.1365-3040.2011.02340.x (2011).

3 Dwyer, S. A., Ghannoum, O., Nicotra, A. & Von Caemmerer, S. High temperature acclimation of C_4 photosynthesis is linked to changes in photosynthetic biochemistry. *Plant Cell Environ* **30**, 53-66, doi:10.1111/j.1365-3040.2006.01605.x (2007).

4 Leuning, R. Temperature dependence of two parameters in a photosynthesis model. *Plant Cell Environ* **25**, 1205-1210, doi:DOI 10.1046/j.1365-3040.2002.00898.x (2002).

5 Heckmann, D. *et al.* Predicting C_4 Photosynthesis Evolution: Modular, Individually Adaptive Steps on a Mount Fuji Fitness Landscape. *Cell* **153**, 1579-1588, doi:http://dx.doi.org/10.1016/j.cell.2013.04.058 (2013).

6 Kubien, D. S., Whitney, S. M., Moore, P. V. & Jesson, L. K. The biochemistry of Rubisco in *Flaveria*. *Journal of experimental botany* **59**, 1767 (2008).

7 Ku, M. S. B. *et al.* Photosynthetic and photorespiratory characteristics of *Flaveria* species. *Plant Physiology* **96**, 518 (1991).

8 Massad, R. S., Tuzet, A. & Bethenod, O. The effect of temperature on C_4-type leaf photosynthesis parameters. *Plant Cell Environ* **30**, 1191-1204, doi:10.1111/j.1365-3040.2007.01691.x (2007).

9 Chen, D. X., Coughenour, M. B., Knapp, A. K. & Owensby, C. E. Mathematical Simulation of C_4 Grass Photosynthesis in Ambient and Elevated CO2. *Ecol Model* **73**, 63-80, doi:Doi 10.1016/0304-3800(94)90098-1 (1994).

10 Tamimi, A., Rinker, E. B. & Sandall, O. C. Diffusion-Coefficients for Hydrogen-Sulfide, Carbon-Dioxide, and Nitrous-Oxide in Water over the Temperature-Range 293-368-K. *J Chem Eng Data* **39**, 330-332, doi:DOI 10.1021/je00014a031 (1994).

11 Zhu, X.-G., de Sturler, E. & Long, S. P. Optimizing the distribution of resources between enzymes of carbon metabolism can dramatically increase photosynthetic rate: a numerical simulation using an evolutionary algorithm. *Plant Physiol* **145**, 513-526, doi:10.1104/pp.107.103713 (2007).

12 R: A Language and Environment for Statistical Computing (R Foundation for Statistical Computing, 2017).

13 The NLopt nonlinear-optimization package.

14 Yin, X. Y. & Struik, P. C. The energy budget in C_4 photosynthesis: insights from a cell-type-specific electron transport model. *New Phytologist* **218**, 986-998, doi:10.1111/nph.15051 (2018).

15 von Caemmerer, S. *Biochemical models of leaf photosynthesis*. (Csiro Publishing, 2000).

16 Bernacchi, C. J., Pimentel, C. & Long, S. P. *In vivo* temperature response functions of parameters required to model RuBP-limited photosynthesis. *Plant Cell Environ* **26**, 1419-1430, doi:DOI 10.1046/j.0016-8025.2003.01050.x (2003).

17 Kramer, D. M. & Evans, J. R. The Importance of Energy Balance in Improving Photosynthetic Productivity. *Plant Physiology* **155**, 70-78, doi:10.1104/pp.110.166652 (2011).

18 Amthor, J. S. From sunlight to phytomass: on the potential efficiency of converting solar radiation to phyto-energy. *New Phytologist* **188**, 939-959, doi:10.1111/j.1469-8137.2010.03505.x (2010).

19 Siebke, K., von Caemmerer, S., Badger, M. & Furbank, R. T. Expressing an *RbcS* antisense gene in transgenic *Flaveria bidentis* leads to an increased quantum requirement for CO_2 fixed in photosystems I and II. *Plant Physiology* **115**, 1163-1174 (1997).

20 Kanai, R. & Edwards, G. E. in *C_4 plant biology* (eds Rowan F Sage & Russell K Monson) 49-87 (Academic press, Toronto, ON, Canada, 1999).

21 Ogren, E. & Evans, J. R. Photosynthetic Light-Response Curves. 1. The Influence of CO_2 Partial-Pressure and Leaf Inversion. *Planta* **189**, 182-190, doi:Doi 10.1007/Bf00195075 (1993).
